# Heuristic satisficing inferential decision making in human and robot active perception

**DOI:** 10.3389/frobt.2024.1384609

**Published:** 2024-11-12

**Authors:** Yucheng Chen, Pingping Zhu, Anthony Alers, Tobias Egner, Marc A. Sommer, Silvia Ferrari

**Affiliations:** ^1^ Sibley School of Mechanical and Aerospace Engineering, Cornell University, Ithaca, NY, United States; ^2^ College of Engineering and Computer Sciences, Marshall University, Huntington, IN, United States; ^3^ Department of Biomedical Engineering (BME), Duke University, Durham, NC, United States; ^4^ Center fir Cognitive Neuroscience, Duke Institute for Brain Sciences, Duke University, Durham, NC, United States

**Keywords:** satisficing, heuristics, active perception, human, studies, decision-making, treasure hunt, sensor

## Abstract

Inferential decision-making algorithms typically assume that an underlying probabilistic model of decision alternatives and outcomes may be learned *a priori* or online. Furthermore, when applied to robots in real-world settings they often perform unsatisfactorily or fail to accomplish the necessary tasks because this assumption is violated and/or because they experience unanticipated external pressures and constraints. Cognitive studies presented in this and other papers show that humans cope with complex and unknown settings by modulating between near-optimal and satisficing solutions, including heuristics, by leveraging information value of available environmental cues that are possibly redundant. Using the benchmark inferential decision problem known as “treasure hunt”, this paper develops a general approach for investigating and modeling active perception solutions under pressure. By simulating treasure hunt problems in virtual worlds, our approach learns generalizable strategies from high performers that, when applied to robots, allow them to modulate between optimal and heuristic solutions on the basis of external pressures and probabilistic models, if and when available. The result is a suite of active perception algorithms for camera-equipped robots that outperform treasure-hunt solutions obtained via cell decomposition, information roadmap, and information potential algorithms, in both high-fidelity numerical simulations and physical experiments. The effectiveness of the new active perception strategies is demonstrated under a broad range of unanticipated conditions that cause existing algorithms to fail to complete the search for treasures, such as unmodelled time constraints, resource constraints, and adverse weather (fog).

## 1 Introduction

Rational inferential decision-making theories obtained from human or robot studies to date assume that a model me be used either off-line or on-line in order to compute satisficing strategies that maximize appropriate utility functions and/or satisfy given mathematical constraints (Simon, 1955; Herbert, 1979; Caplin and Glimcher, 2014; Nicolaides, 1988; Simon, 2019). When a probabilistic world model is available, for example, methods such as optimal control, cell decomposition, probabilistic roadmaps, and maximum utility theories, may be applied to inferential decision-making problems such as robot active perception, planning, and feedback control (Fishburn, 1981; Lebedev et al., 2005; Scott, 2004; Todorov and Jordan, 2002; Ferrari and Wettergren, 2021; Latombe, 2012; LaValle, 2006). In particular, active perception, namely, the ability to plan and select behaviors that optimize the information extracted from the sensor data in a particular environment, has broad and extensible applications in robotics that also highlights human abilities to make decisions when only partial or imperfect information is available.

Many “model-free” reinforcement learning (RL) and approximate dynamic programming (ADP) approaches have also been developed on the basis of the assumption that a partial or imperfect model is available in order to predict the next system state and/or “cost-to-go”, and optimize the immediate and potential future rewards, such as information value (Bertsekas, 2012; Si et al., 2004; Powell, 2007; Ferrari and Cai, 2009; Sutton and Barto, 2018; Wiering and Van Otterlo, 2012; Abdulsaheb and Kadhim, 2023). Given the computational burden carried by learning-based methods, various approximations have also been proposed. For instance, approximate dynamic programming (ADP) methods have been developed based on the assumption that a partial or imperfect model is available to predict the next system state and/or “cost-to-go.” These methods aim to optimize immediate and potential future rewards, such as information value (Bertsekas, 2012; Si et al., 2004; Powell, 2007; Ferrari and Cai, 2009; Sutton and Barto, 2018; Wiering and Van Otterlo, 2012), typically also exploiting world models available *a priori* in order to predict the next world state.

Other machine learning (ML) and artificial intelligence (AI) methods can be broadly categorized into two fundamental learning-based approaches. The first approach is deep reinforcement learning (DRL), where models incorporate classical Markov decision process theories and use a human-crafted or data-extracted reward function to train an agent to maximize the probability of gaining the highest reward (Silver et al., 2014; Lillicrap et al., 2015; Schulman et al., 2017). The second approach follows the learning from demonstration paradigm, also known as imitation learning (Chen et al., 2020; Ho and Ermon, 2016). Because of their need for extensive and domain-specific data, data-driven methods are also not typically applicable to situations that cannot be foreseen *a priori*.

Given the ability of natural organisms to cope with uncertainty and adapt to unforeseen circumstances, a parallel thread of development has focused on biologically inspired models, especially for perception-based decision making. These methods are typically computationally highly efficient and include motivational models, which use psychological motivations as incentives for agent behaviors (Lewis and Cañamero, 2016; O’Brien and Arkin, 2020; Lones et al., 2014), cognitive models, which transfer human mental and emotional functions into robots (Vallverdú et al., 2016; Martin-Rico et al., 2020). The implementation of cognitive models are usually in the form of heuristics, and their applications range from energy level maintenance (Batta and Stephens, 2019) to domestic environment navigation (Kirsch, 2016).

Humans have also been shown to use internal world models for inferential decision-making whenever possible, a characteristic first referred to as “substantial rationality” in (Simon, 1955; Herbert, 1979). As also shown by the human studies on passive and active satisficing perception presented in this paper, given sufficient data, time, and informational resources, a globally rational human decision-maker uses an internal model of available alternatives, probabilities, and decision consequences to optimize both decision and information value in what is known as a “small-world” paradigm (Savage, 1972). In contrast, in “large-world” scenarios, decision-makers face environmental pressures that prevent them from building an internal model or quantifying rewards, because of pressures such as missing data, time and computational power constraints, or sensory deprivation, yet still manage to complete tasks by using “bounded rationality” (Simon, 1997). Under these circumstances, optimization-based methods may not only be infeasible, returning no solution, but also cause disasters resulting from failing to take action (Gigerenzer and Gaissmaier, 2011). Furthermore, Simon and other psychologists have shown that humans can overcome these limitations in real life via “satisficing decisions” that modulate between near-optimal strategies and the use of heuristics to gather new information and arrive at fast and “good-enough” solutions to complete relevant tasks.

To develop satisficing solutions for active robot perception, herein, we consider here the class of sensing problems known as treasure hunt (Ferrari and Cai, 2009; Cai and Ferrari, 2009; Zhang et al., 2009; Zhang et al., 2011). The mathematical model of the problem, comprised of geometric and Bayesian network descriptions demonstrated in (Ferrari and Wettergren, 2021; Cai and Ferrari, 2009), is used to develop a new experimental design approach that ensures humans and robots experience the same distribution of treasure hunts in any given class, including time, cost, and environmental pressures inducing satisficing strategies. This novel approach enables not only the readily comparison of the human-robot performance but also the generalization of the learned strategies to any treasure hunt problem and robotic platform. Hence, satisficing strategies are modeled using human decision data obtained from passive and active satisficing experiments, ranging from desktop to virtual reality human studies sampled from the treasure hunt model. Subsequently, the new strategies are demonstrated through both simulated and physical experiments involving robots under time and cost pressures, or subject to sensory deprivation (fog).

The treasure hunt problem under pressure, formulated in Section 2. and referred to as satisficing treasure hunt herein, is an extension of the robot treasure hunt presented in Cai and Ferrari (2009); Zhang et al. (2009), which introduces motion planning and inference in the search for Spanish treasures originally used in Simon and Kadane (1975) to investigate satisficing decisions in humans. Whereas the search for Spanish treasures amounts to searching a (static) decision tree with hidden variables, the robot treasure hunt involves a sensor-equipped robot searching for targets in an obstacle-populated workspace. As shown in Ferrari and Wettergren (2021) and references therein, the robot treasure hunt paradigm is useful in many mobile sensing applications involving multi-target detection and classification. In particular, the problem highlights the coupling of action decisions that change the physical state of the robot (or decision-maker) with test decisions that allow the robot to gather information from the targets via onboard sensors. In this paper, the satisficing treasure hunt is introduced to investigate and model human satisficing perception strategies under external pressures in passive and active tasks, first via desktop simulations and then in the Duke immersive Virtual Environment (DiVE) (Zielinski et al., 2013), as shown in Supplementary Figure S1.

To date, substantial research has been devoted to solving treasure hunt problems for many robots/sensor types, in applications as diverse as demining infrared sensors and underwater acoustics, under the aforementioned “small-world” assumptions (Ferrari and Wettergren, 2021). Optimal control and computational geometry solution approaches, such as cell decomposition (Cai and Ferrari, 2009), disjunctive programming (Swingler and Ferrari, 2013), and information roadmap methods (IRM) (Zhang et al., 2009), have been developed for optimizing robot performance by minimizing the cost of traveling through the workspace and processing sensor measurements, while maximizing the sensor rewards such as information gain. All these existing methods assume prior knowledge of sensor performance and of the workspace, and are applicable when the time and energy allotted to the robot are adequate for completing the sensing task. Information-driven path planning algorithm integrated with online mapping, developed in Zhu et al. (2019); Liu et al. (2019); Ge et al. (2011), have extended former treasure hunt solutions to problems in which a prior model of the workspace is not available and must be obtained online. Optimization-based algorithms have also been developed for fixed end-time problems with partial knowledge of the workspace, on the basis of the assumption that a probabilistic model of the information states and unlimited sensor measurements are available (Rossello et al., 2021). This paper builds on this previous work to develop heuristic strategies applicable when uncertainties cannot be learned or mathematically modeled in closed form, and the presence of external pressures might prevent task completion, e.g., adverse weather or insufficient time/energy.

Inspired by previous findings on human satisficing heuristic strategies (Gigerenzer and Gaissmaier, 2011; Gigerenzer, 1991; Gigerenzer and Goldstein, 1996; Gigerenzer, 2007; Oh et al., 2016), this paper develops, implements, and compares the performance between existing treasure hunt algorithms and human participants engaged in the same sensing tasks and experimental conditions by using a new design approach. Subsequently, human strategies and heuristics outperforming existing state-of-the-art algorithms are identified and modeled from data in a manner that can be extended to any sensor-equipped autonomous robot. The effectiveness of these strategies is then demonstrated with camera-equipped robots via high-fidelity simulations as well as physical laboratory experiments. In particular, human heuristics are modeled by using the “three building blocks” structure for formalizing general inferential heuristic strategies presented in Gigerenzer and Todd (1999). The mathematical properties of heuristics characterized by this approach are then compared with logic and statistics, according to the rationale in Gigerenzer and Gaissmaier (2011).

Three main classes of human heuristics for inferential decisions exist: recognition-based decision-making (Ratcliff and McKoon, 1989; Goldstein and Gigerenzer, 2002), one-reason decision-making (Gigerenzer, 2007; Newell and Shanks, 2003), and trade-off heuristics (Lichtman, 2008). Although categorized by respective decision mechanisms, these classes of human heuristics have been investigated in disparate satisficing settings, thus complicating the determination of which strategies are best equipped to handle different environmental pressures. Furthermore, existing human studies are typically confined to desktop simulations and do not account for action decisions pertaining to physical motion and path planning in complex workspaces. Therefore, this paper presents a new experimental design approach (Section 3) and tests in human participants to analyze and model satisficing active perception strategies (Section 7) that are generalizable and applicable to robot applications, as shown in Section 8.

The paper also presents new analysis and modeling studies of human satisficing strategies in both passive and active perception and decision-making tasks (Section 3). For passive tasks, time pressure on inference is introduced to examine subsequent effects on human decision-making in terms of decision model complexity and information gain. The resulting heuristic strategies (Section 5) extracted from human data demonstrate adaptability to varying time pressure, thus enabling inferential decision-making to meet decision deadlines. These heuristics significantly reduce the complexity of target feature search from an exhaustive search $O\left(2^{n}\right)$ to O(nlog(n)+n) , where n is the number of target features. Additionally, they exhibit superior classification performance when compared to optimizing strategies that utilize all target features for inference (Section 6), demonstrating the less-can-be-more effect (Gigerenzer and Gaissmaier, 2011).

For active tasks, when the sensing capabilities are significantly hindered, such as in adverse weather conditions, human strategies are found to amount to highly effective heuristics that can be modeled as shown in Section 7, and generalized to robots as shown in Section 8. The human strategies discovered from human studies are implemented on autonomous robots equipped with vision sensors and compared with existing planning methods (Section 8) through simulations and physical experiments in which optimizing strategies fail to complete the task or exhibit very poor performance. Under information cost pressure, a decision-making strategy developed using mixed integer nonlinear program (MINLP) (Cai and Ferrari, 2009; Zhang et al., 2009) was found to outperform existing solutions as well as human strategies (Section 8). By complementing the aforementioned heuristics, the MINLP optimizing strategies provide a toolbox for active robot perception under pressures that is verified both in experiments and simulations.

## 2 Treasure hunt problem formulation

This paper considers the active perception problem known as treasure hunt, in which a mobile information-gathering agent, such as a human or an autonomous robot, must find and localize all important targets, referred to as *treasures*, in an unknown workspace $W\subset R^{3}$ . The number of possible treasures or targets, r , is unknown *a priori*, and each target i may constitute a treasure or another object, such as a clutter or false alarm, such that its classification may be represented by a random and discrete hypothesis variable $Y_{i}$ with finite range $Y=\left\{y_{j}\;|\;j\in J\right\}$ , where $y_{j}$ represents the j th category of $Y_{i}$ . While $Y_{i}$ is hidden or non-observable, it may be inferred from $p_{i}\in Z$ observed features among a set of n discrete random variables $X_{i}=\left\{X_{i,1},\ldots ,X_{i,n}\right\}$ , and the l th (1≤l≤n) feature has a finite range $X_{l}=\left\{x_{l,j}\;|\;j\in N\right\}$ [see (Ferrari and Cai, 2009; Zhang et al., 2011; Ferrari and Vaghi, 2006) for more details]. At the onset of the search, $X_{i}$ and $Y_{i}$ are assumed unknown for all targets, as are the number of targets and treasures present in W . Thus, the agent must first navigate the workspace to find the targets and, then, observe their features to infer their classification.

All r targets are fixed, at unknown positions $x_{1},\ldots ,x_{r}\in W$ , and must be detected, observed, and classified using onboard sensors with bounded field-of-view (FOV) (Ferrari and Wettergren, 2021):

Definition 2.1 (Field-of-view (FOV)) For a sensor characterized by a dynamic state, in a workspace $W\subset R^{3}$ , the FOV is defined as a closed and bounded subset S⊂W such that a target feature $X_{i,l}$ may be observed at any point $x_{i}\in S$ .

In order to obtain generalizable strategies for camera-equipped robots, in both human and robot studies knowledge of the targets is acquired, at a cost, through vision, and the sensing process is modeled by a probabilistic Bayesian network learned from data (Ferrari and Wettergren, 2021).

**FIGURE 1:**
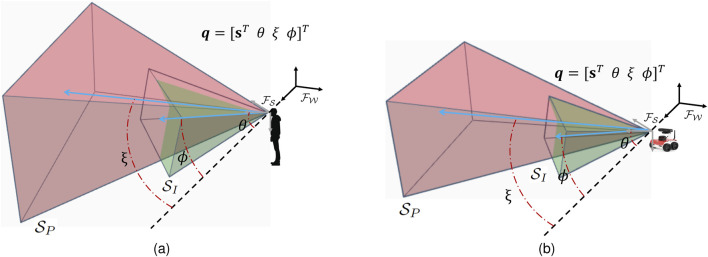
Human **(A)** and robot **(B)** state, configuration, and passive and active sensor FOVs.

Although the approach can be easily extended to other sensor configurations, in this paper it is assumed that the information-gathering agent is equipped with one passive sensor for obstacle/target collision avoidance and localization, with FOV denoted by $S_{P}$ , and one active sensor for target inference and classification, with FOV denoted by $S_{I}$ (Figure 1B). In human studies, the same passive/active configuration is implemented via virtual reality (VR) wand/joystick and goggles, and by measuring and constraining the human FOV, as shown in Figure 1A. Furthermore, the workspace is populated with q known fixed, rigid, and opaque objects $B_{1},\ldots ,B_{q}\subset W$ that constitute obstacles as well as occlusions. Therefore, in order to observe the targets, the agent must navigate in W avoiding both collisions and occluded views, according to the following line of sight (LOS) visibility constraint:

Definition 2.2 (Line of sight) Given the sensor position s∈W , a target at x∈W is occluded by an object B⊂W if and only if,

$$L\left(s,x\right)\cap B\neq \emptyset$$

where L(s,x)={(1−γ)s+γx | γ∈[0,1]} .

Let $F_{W}$ denote an inertial frame embedded in W , and I denote the geometry of the agent body. The motion of the agent relative to the workspace can then be described by the position and orientation of a body frame $F_{S}$ , embedded in the agent, relative to $F_{W}$ . Thus, the state of the information-gathering agent at $t_{k}$ can be described by the vector $q_{k}={\left[s_{k}^{T}\;\theta_{k}\;\xi_{k}\;\phi_{k}\right]}^{T}$ , where $s_{k}$ represents the inertial position of the information-gathering agent in W , $\theta_{k}\in S^{1}$ is the orientation of the agent, and $\xi_{k}\in \left[\xi_{l},\xi_{u}\right]$ and $\phi_{k}\in \left[\phi_{l},\phi_{u}\right]$ are preferred sensing directions of the “passive” and “active” FOVs, respectively. In addition, $\xi_{l},\xi_{u}$ and $\phi_{l},\phi_{u}$ bound the preferred sensing directions for $S_{P}$ and $S_{I}$ with respect to the information-gathering agent body. By this approach it is possible to model FOVs able to move with respect to the agent body, as required by the motion of the human head or pan-tilt-zoom cameras (Figure 1).

Obstacle avoidance is accomplished by ensuring that the agent configuration, defined as $t_{k}={\left[s_{k}^{T}\;\theta_{k}\right]}^{T}$ , remains in free configuration space at all times. Let C represent all possible agent configurations, and ${CB}_{j}=\left\{t\in C|I\left(t\right)\cap B_{j}\neq \emptyset \right\}$ denote the C-obstacle associated with object $B_{j}$ [defined in Ferrari and Wettergren (2021) and references therein). Then, the free configuration space is the space of configurations that avoid collisions with the obstacles or, in other words, that are the complement of all C-obstacle regions in C , i.e., $C_{\text{free}}=\left\{C\backslash {⋃}_{j=1}^{q}{CB}_{j}\right\}$ .

According to directional visibility theory (Gemerek et al., 2022), the subset of the free space at which a target is visible by a sensor in the presence of occlusions can defined as follows:

Definition 2.3 (Target Visibility Region) For a sensor with FOV $S_{P}\subset W$ , in the presence of q occlusions $B_{j}\left(j=1,\ldots ,q\right)$ a target at $x_{i}\in W$ is visible within the target visibility region that satisfies both FOV and LOS conditions, i.e.,:

$${TV}_{i}=\left\{t\in C_{\text{free}}\;|\;x_{i}\in S_{P},L\left(s,x_{i}\right)\cap B_{j}=\emptyset ,\forall j\right\}$$

It follows that multiple targets are visible to the sensor in the intersection of multiple visibility regions defined as Gemerek et al. (2022):

Definition 2.4 (Set Visibility Region) Given a set of r target-visibility regions $\left\{{TV}_{i}\;|\;i\in \left\{1,2,\ldots ,r\right\}\right\}$ , let S⊆{1,2,…,r} represent the set of target indices of two or more intersecting regions, such that the following holds ${⋂}_{i\in S}TV_{i}\neq \emptyset$ . Then, the set visibility region of target i is defined as

$$V_{S}=\left\{\underset{i\in S}{⋂}TV_{i}\;|\;S\subseteq \left\{1,2,\ldots ,r\right\}\right\}$$

**FIGURE 2:**
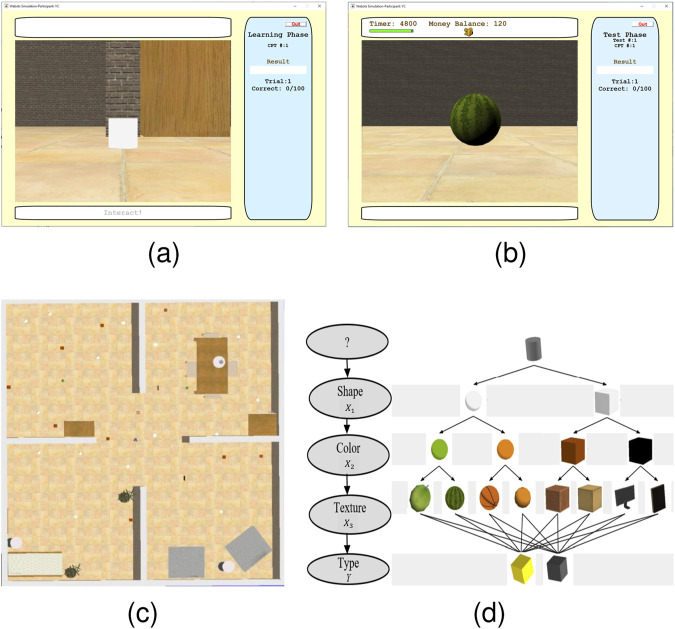
First-person view in training phase without prior target feature revealed **(A)** and with feature revealed by a participant **(B)** in the Webots®workspace **(C)** and target features encoded in a BN structure with ordering constraints **(D)**.

Similarly, after a target i is detected and localized, the agent may observe the target features using the active sensor with FOV $S_{I}$ provided $x_{i}\in S_{I}\left(q\right)$ and $L\left(s,x_{i}\right)\cap B_{j}=\emptyset ,\;\;1\leq j\leq q$ . In order to explore the tradeoff of information value and information cost in inferential decisions, use of the active sensor is associated with an information cost $J\left(t_{k}\right)$ that may reflect the use of processing power, data storage, and/or need for covertness. Then, the information-gathering agent, must make a deliberate decision to observe one or more target features prior to obtaining the corresponding measurement, which may consist of an image or raw measurement data from which feature $X_{i}$ may be extracted. For simplicity, measurement errors are assumed negligible but they may be easily introduced following the approach in [10, Chapter 9]. Then, the goal of the treasure hunt is to infer the hypothesis variable $Y_{i}$ from $X_{i}$ , i=1,2,… , using a probabilistic measurement model $P\left(Y_{i},X_{i,1},\ldots ,X_{i,n}\right)$ (Ferrari and Cai, 2009). The measurement model, chosen here as a Bayesian network (BN) (Figure 2D), consists of a probabilistic representation of the relationship between the observed target features and the target classification that may be learned from expert knowledge or prior training data as shown in [10, Chapter 9]. Importantly, because the agent may not have the time and/or resources to observe all target features, classification may be performed from a sequence of partial observations.

Target features are observed through test decisions made by the information-gathering agent, which result into soft or hard evidence for the probabilistic model $P\left(Y_{i},X_{i,1},\ldots ,X_{i,n}\right)$ (Jensen and Nielsen, 2007). Let $u\left(t_{k}\right)\in U_{k}$ denote at time $t_{k}$ test decision chosen from the set of all admissible tests $U_{k}\subset U$ . The set $U=\left\{\vartheta_{c},\vartheta_{s},\vartheta_{un}\right\}$ consists of all test decisions, where $\vartheta_{c}$ and $\vartheta_{s}$ represent the decisions to continue or stop observing target features, and $\vartheta_{un}$ represents the decision to not observe any feature. The test decision $u\left(t_{k}\right)$ generates a measurement variable at time step $t_{k+1}$ ,

$$z\left(t_{k+1}\right)=x_{i,l},\;1\leq i\leq r,\;1\leq l\leq n,\;x_{i,l}\in X_{l}$$

observed after paying the information cost $J\left(t_{k}\right)\in Z$ , which is modeled as cumulative number of observed features up to $t_{k}$ . When the measurement budget R is finite, it may not be exceeded by the agent and, thus, the treasure hunt problem must be solved subject to the hard constraint

$$J\left(t_{k}\right)\leq R.$$

Action decisions modify the state of the world and/or information-gathering agent (Jensen and Nielsen, 2007). In the treasure hunt problem, action decisions are control inputs that decide the position and orientation of the agent and of the FOVs $S_{P}$ and $S_{I}$ . Let $a\left(t_{k}\right)\in A_{k}$ denote an action decision chosen at time $t_{k}$ from set $A_{k}$ of all admissible actions. The agent motion can then be described by a causal model as the following difference equation,

$$q_{k+1}=f\left[q_{k},a\left(t_{k}\right),t_{k}\right]$$

where f[⋅] is obtained by modeling the agent dynamics.

Then, an active perception strategy consists of a sequence of action and test decisions that allow the agent to search the workspace and obtain measurements from targets distributed therein, as follows:

Definition 2.5 (Inferential Decision Strategy) An active inferential decision strategy is a class of admissible policies that consists of a sequence of functions,

$$\sigma =\left\{\pi_{0},\pi_{1},\ldots ,\pi_{T}\right\}$$

where $\pi_{k}$ maps all past information-gathering agent states, test variables, action and test decisions into admissible action and test decisions,

$$\begin{array}{cc} \left\{a\left(t_{k}\right),u\left(t_{k}\right)\right\} & =\pi_{k}\left[q_{0},a\left(t_{1}\right),u\left(t_{1}\right),z\left(t_{1}\right),J\left(t_{1}\right),q_{1},\right.\\ & \;\left.\ldots ,a\left(t_{k-1}\right),u\left(t_{k-1}\right),z\left(t_{k-1}\right),J\left(t_{k-1}\right),q_{k-1}\right] \end{array}$$

such that $\pi_{k}\left[\cdot \right]\in \left\{A_{k},U_{k}\right\}$ , for all k=1,2,…,T .

Based on all the aforementioned definitions, the problem is formulated as follows:

Problem 1 (Satisficing Treasure Hunt)

Given an initial state $q_{0}$ and the satisificing aspiration level of total information value Δ , the satisficing treasure hunt problem consists of finding an active inferential decision making strategy, σ , over a known and finite time horizon (0,T] , such that the cumulative information value collected from all observed features is no less than Δ ,

$$\overset{r}{\underset{i=1}{\sum }}\left[1\left(\exists k,x_{i}\in S_{I}\left(q_{k}\right)\wedge L\left(s_{k},x_{i}\right)\cap B_{j},\forall j\right)I\left(Y_{i};X_{i}\right)\right]\geq \Delta$$ (1)

where

$$q_{k+1}=f\left[q_{k},a\left(t_{k}\right),t_{k}\right]$$ (2)

$${\hat{y}}_{i}={\mathrm{arg max}}_{y\in Y}P\left(Y_{i}=y,X_{i,1},\ldots ,X_{i,n}\right)$$ (3)

$$I\left(Y_{i};X_{i}\right)=H\left(Y_{i}\right)-H\left(Y_{i}\;|\;X_{i}\right)$$ (4)

$$J\left(t_{T}\right)\leq R$$ (5)

i=1,2,…,r,1≤k≤T (6)

j=1,2,…,q (7)

An optimal search strategy makes use of the agent motion model (Equation 2), measurement model (Equation 3) and knowledge of the workspace W to maximize the information value while minimizing the distance traveled and the cumulative information cost (Ferrari and Wettergren, 2021). A feasible search strategy may use all or part of the available models of the environment and targets, or knowledge of prior states and decisions to produce a sequence of action and test decisions that satisfy the objective (Equation 1) by the desired end time $t_{T}$ .

## 3 Human satisficing studies

Human strategies and heuristics for active perception are modeled and investigated by considering two classes of satisficing treasure hunt problems, referred to as passive and active experiments. Passive satisficing experiments focus on treasure hunt problems in which information is presented to the decision maker who passively observes features needed to make inferential decisions. Active satisficing experiments allow the decision maker to control the amount of information gathered in support of inferential decisions. Additionally, treasure hunt problems with both static and dynamic robots are considered in order to compare with and extend previous satisficing studies, evolving human studies traditionally conducted on a desktop (Oh et al., 2016; Toader et al., 2019; Oh-Descher et al., 2017) to ambulatory human studies in virtual reality that parallel mobile robots applications (Zielinski et al., 2013).

Previous cognitive psychology studies showed that the urgency to respond (Cisek et al., 2009) and the need for fast decision-making (Oh et al., 2016) significantly affect human decision evidence accumulation, thus leading to the use of heuristics in solving complex problems. Passive satisficing experiments focus on test decisions, which determine the evidence accumulation of the agent based on partial information under “urgency”. Inspired by satisficing searches for Spanish treasures with feature ordering constraints (Simon and Kadane, 1975), active satisficing includes both test and action decisions, which change not only the agent’s knowledge and information about the world but also its physical state. Because information gathering by a physical agent such as a human or robot is a causal process (Ferrari and Wettergren, 2021), feature ordering constraints are necessary in order to describe the temporal nature of information discovery.

Both passive and active satisficing human experiments comprise a training phase and a test phase that are also similarly applied in the robot experiments in Sections 6–8. During the training phase, human participants learn the validity of target features in determining the outcome of the hypothesis variable. They receive feedback on their inferential decisions to aid in their learning process. During the test phase, pressures are introduced, and action decisions are added for active tasks. Importantly, during the test phase, no performance feedback or ground truth is provided to human participants (or robots).

### 3.1 Passive satisficing task

The passive satisficing experiments presented in this paper adopted the passive treasure hunt problem, shown in Supplementary Figure S2 and related to the well-known weather prediction task (Gluck et al., 2002; Lagnado et al., 2006; Speekenbrink et al., 2010). The problem was first proposed in Oh et al. (2016) to investigate the cognitive processes involved in human test decisions under pressure. In view of its passive nature, the experimental platform of choice consisted of a desktop computer used to emulate the high-paced decision scenarios, and to encourage the human participants to focus on cue(feature) combination rather than memorization (Oh et al., 2016; Lamberts, 1995).

The stimuli presented on a screen were precisely controlled, ensuring consistency across participants and minimizing distractions from irrelevant objects or external factors (Garlan et al., 2002; Lavie, 2010). In each task, participants were presented with two different stimuli from which to select the “treasure” before the total time, $t_{T}$ , at one’s disposal has elapsed (time pressure). The treasures are hidden but correlated with the visual appearance of the stimulus, and the underlying probabilities must be learned by trial and error during the training phase. Each stimulus is characterized by four binary cues or “features”, namely, color $\left(X_{1}\right)$ , shape $\left(X_{2}\right)$ , contour $\left(X_{3}\right)$ , and line orientation $\left(X_{4}\right)$ , illustrated in the table in Supplementary Figure S2. The goal of this passive satisficing task is to find all treasures among stimuli that are presented on the screen or, in other words, to infer a binary hypothesis variable Y , with range $Y=\left\{y_{1},y_{2}\right\}$ , where $y_{1}=$ “treasure” and $y_{2}=$ “not treasure”. The task is passive by design because the participant cannot control the information displayed in order to aid his/her decisions.

During the training phase, each (human) participant performed 240 trials in order to learn the relationship between features, $X=\left\{X_{1},X_{2},X_{3},X_{4}\right\}$ , and the hypothesis variable Y . After the training phase, participants were divided into two groups. The first group underwent a moderate time pressure (TP) experiment and was tested against two datasets, each consisting of 120 trials. Participants were required to make decisions within a response time $t_{T}=750$ ms, which allowed ample time to ponder on the features presented and how they related to the treasure. The second group underwent an intense TP experiment, with a response time of only $t_{T}=500$ ms. Participants in this group also encountered two datasets, each containing 120 trials. A more detailed description of the experiment, including redundant features, and human subject procedures that informed, among other parameters, the number of trials can be found in Oh et al. (2016). Subsequently, the task was modified to develop a number of active satisficing treasure hunts in which information about the treasures had to be obtained by navigating a complex environment, as explained in the next section.

As shown in Table 1, the relevant statistics for passivesatisficing experiments are summarized in the upper part. Similarly, the statistics for active satisficing experiments are presented in the lower part, where the human participants are allowed to move in an environment and choose the interaction order with the targets. The statistics correspond to three conditions: “No Pressure”, “Info Cost Pressure”, and “Sensory Deprivation”. These pressure conditions will be introduced in detail in Section 3.2.

**TABLE 1:** Experiment conditions and trials.

Experiment type	Pressure condition	Number of participants	Number of training targets for each participant	Number of test trials for each participant
Passive Satisficing	NP	48	240	120
	Moderate TP	48	240	120
	Intense TP	48	240	120
Active Satisficing	No Pressure	6	100	3
	Info Cost Pressure	6	100	3
	Sensory Deprivation	6	100	3

### 3.2 Active satisficing treasure hunt task

The satisficing treasure hunt task is an ambulatory study in which participants must navigate a complex environment populated with a number of obstacles and objects in order to first find a set of targets (stimuli) and, then, determine which are the treasures. Additionally, once the targets are inside the participant’s FOV, features are displayed sequentially to him/her only after paying cost for the information requested. The ordering constraints (illustrated in Figure 2D) allow for the study of information cost and its role in the decision making process by which the task is to be performed not only under time pressure but also a fixed budget. Thus, the satisficing treasure hunt allows not only to investigate how information about a hidden variable (treasure) is leveraged, but also how humans mediate between multiple objectives such as obstacle avoidance, limited sensing resources, and time constraints. Participants must, therefore, search and locate the treasures without any prior information on initial target features, target positions, or workspace and obstacle layout.

In order to utilize a controlled environment that can be easily changed to study all combinations of features, target/obstacle distributions, and underlying probabilities, the active satisficing treasure hunt task was developed and conducted in a virtual reality environment known as the DiVE (Zielinski et al., 2013). By this approach different experiments were designed and easily modified so as to investigate different difficulty levels and provide the human participants repeatable, well-controlled, and immersive experience of acquiring and processing information to generate behavior (Van Veen et al., 1998; Pan and Hamilton, 2018; Servotte et al., 2020). The DiVE consists of a 3 m × 3 m × 3 m stereoscopic rear projected room with head and hand tracking, allowing participants to interact with a virtual environment in real-time (Zielinski et al., 2013). By developing a new interface between the DiVE and the robotic software ${Webots}^{Ⓡ}$ , this research was able to readily introduce humans within the same environments designed for humans, and *vice versa*, according to the BN model of the desired treasure hunt task. The structure of the BN used for the human/robot treasure hunt perception task is plotted in Figure 2D. The BN parameters, not shown for brevity, were varied across trials to obtain a representative dataset from the human study from which mathematical models of human decision strategies could be learned and validated.

Six human participants were trained and given access to the DiVE for a total of fifty-four trials with the objective to model aspects of human intelligence that outperform existing robot strategies. The number of trials and participants is adequate to the scope of the study which was not to learn from a representative sample of the human population, but to extract inferential decision making strategies generalizable to treasure hunt robot problems. Besides manageable in view of the high costs and logistical challenges associated with running DiVE experiments, the size of the resulting dataset was also found to be adequate to varying all of the workspace and target characteristics across experiments, similarly to the studies in Ziebart et al. (2008); Levine et al. (2011). Moreover, through the VR googles and environment, it was possible to have precise and controllable ground truth not only about the workspace, but also about the human FOV, $S_{P}$ , within which the human could observe critical information such as targets, features, and obstacles.

A mental model of the relationship between target features and classification was first learned by the human participants during 100 stationary training sessions (Figures 2A, B) in which the target features (visual cues), comprised of shape $\left(X_{1}\right)$ , color $\left(X_{2}\right)$ , and texture $\left(X_{3}\right)$ , followed by the target classification Y , where $Y=\left\{y_{1},y_{2}\right\}$ , were displayed on a computer screen, through the desktop ${Webots}^{Ⓡ}$ simulation shown in Figure 2. Participants were then instructed to search for treasures inside an unknown 10 m × 10 m ${Webots}^{Ⓡ}$ workspace with r=30 targets (Figure 2C), by paying information cost $J\left(t_{k}\right)$ to see the features, $X_{i}=\left\{X_{i,1},X_{i,2},X_{i,3}\right\}$ , of every target (labeled by i ) inside their FOV sequentially over time (test phase). Based on the features observed, which may have included one or more features in the set X , participants were asked to decide which targets were treasures $\left(Y=y_{1}\right)$ or not $\left(Y=y_{2}\right)$ . No feedback about their decisions was provided and, as explained in Section 2, the task had to be performed within a limited budget R and time period $t_{T}$ .

**FIGURE 3:**
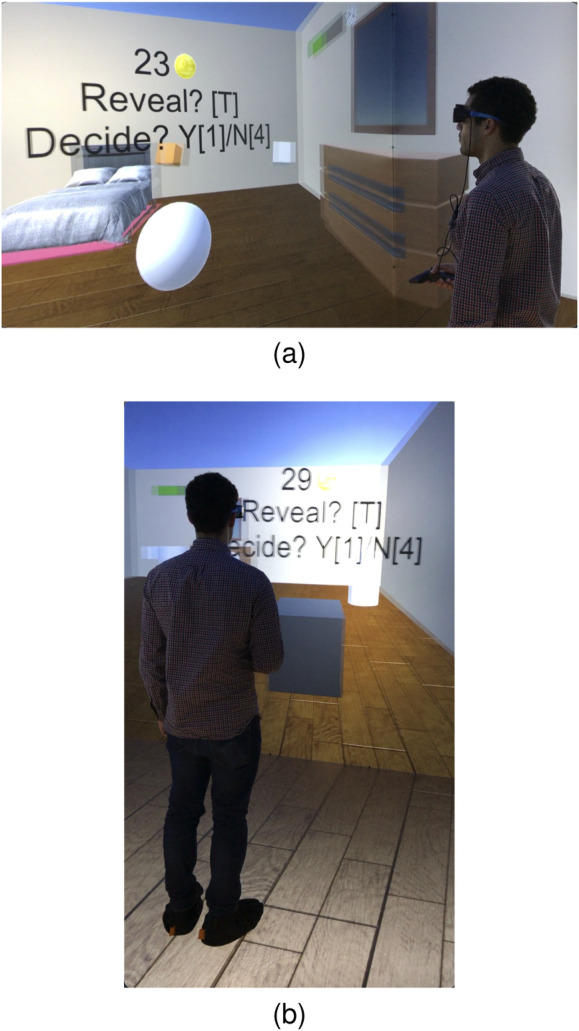
Test phase in active satisficing experiment in DiVE from side view **(A)**, and from rear view **(B)**.

Mobility and ordering feature constraints are both critical to autonomous sensors and robots, because they are intrinsic to how these cyber-physical systems gather information and interact with the world around them. Thanks to the simulation environments and human experiment design presented in this section, we were able to engage participants in a series of classification tasks in which target features were revealed only after paying both a monetary and time cost, similarly to artificial sensors that require both computing and time resources to process visual data. Participants were able to build a mental model built for decision making with the inclusion of temporal constraints during the training phase, according to the BN conditional probabilities (parameters) of each study. By sampling the ${Webots}^{Ⓡ}$ environments from each BN model, selected by the experiment designer to encompass the full range of inference problem difficulty, and by transferring them automatically into VR (Figure 3) the data collected was guaranteed ideally suited for the modeling and generalization of human strategies to robots (Section 7). As explained in the next section, the test phase was conducted under three conditions: no pressure, money pressure, and sensory deprivation (fog).

## 4 External pressures inducing satisficing

Previous work on human satisficing strategies and heuristics illustrated that most humans resort to these approaches for two main reasons, one is computational feasibility and the other is the “less-can-be-more” effect (Gigerenzer and Gaissmaier, 2011). When the search for information and computation costs become impractical for making a truly “rational” decision, satisficing strategies adaptively drop information sources or partially explore decision tree branches, thus accommodating the limitations of computational capacity. In situations in which models have significant deviations from the ground truth, external uncertainties are substantial, or closed-form mathematical descriptions are lacking, optimization on potentially inaccurate models can be risky. As a result, satisficing strategies and heuristics often outperform classical models by utilizing less information. This effect can be explained in two ways. Firstly, the success of heuristics is often dependent on the environment. For example, empirical evidence suggests that strategies such as “take-the-best,” which rely on a single good reason, perform better than classical approaches under high uncertainty (Hogarth and Karelaia, 2007). Secondly, decision-making systems should consider trade-offs between bias and variance, which is determined by model complexity (Bishop and Nasrabadi, 2006). Simple heuristics with fewer free parameters have smaller variance than complex statistical models, thus avoiding overfitting to noisy or unrepresentative data, and generalizable across a wider range of datasets (Bishop and Nasrabadi, 2006; Brighton et al., 2008; Gigerenzer and Brighton, 2009).

Motivated by the situations where robots’ mission goals can be severely hindered or completely compromised due to inaccurate environment or sensing models caused by pressures, the paper seeks to emulate aspects of human intelligence under the pressures and study their influence on decisions. The environment pressures include, for example, time pressure (Payne et al., 1988), information cost (Dieckmann and Rieskamp, 2007; Bröder, 2003), cue(feature) redundancy (Dieckmann and Rieskamp, 2007; Rieskamp and Otto, 2006), sensory deprivation, and high risks (Slovic et al., 2005; Porcelli and Delgado, 2017). Cue(feature) redundancy and high risk have been investigated extensively in statistics and economics, particularly in the context of inferential decisions (Kruschke, 2010; Mullainathan and Thaler, 2000). In the treasure hunt problem, sensory deprivation and information cost directly and indirectly influence action decisions, which brings insight how these pressures impact agents’ motion. However, the effects of sensory deprivation on human decisions have not been thoroughly investigated compared to other pressures. Time pressure is ubiquitous in the real world, yet heuristic strategies derived from human behavior are still lacking. Thus, this paper aims to fill this research gap by examining the time pressure, information cost pressure, and sensory deprivation and their effects on decision outcomes.

### 4.1 Time pressure

Assume that a fixed time interval $t_{c}$ is needed to integrate one additional feature into the inference decision-making process. In the meantime, each decision must be made within $t_{T}$ , and $p_{i}$ is the number of observed features for the i th target. The satisficing strategies must adaptively select a subset of the features such that a decision is made within the time constraint

$$p_{i}t_{c}<t_{T},\;i=1,2,\ldots ,r$$

According to the human studies in Oh et al. (2016), the response time of participants in the passive satisficing tasks was measured during the pilot work. The average response time in these tasks was found to be approximately 700 ms. Based on this finding, three time windows were designed to represent different time pressure levels: a 2-s time window was considered without any time pressure; a 750 ms time window was considered moderate time pressure; and a 500 ms time window was considered intense time pressure.

### 4.2 Information cost

The cost of acquiring new information intrinsically makes an agent use fewer features to reach a decision. In Section 2, new information for the i th target is collected through a sequence of $p_{i}$ observed target features. Thus, for all r targets, the information cost is mathematically described as the total number of observed features not exceeding a preset budget R

$$\overset{r}{\underset{i=1}{\sum }}p_{i}\leq R$$

In Section 3.2, the human studies introduce information cost pressure using the parameter R=30 . In the context of the treasure hunt problem, R represents the measurement budget, which limits the number of features that a participant can observe from targets. In this experiment, for example, a total of r=30 targets was used, and an information budget of R=30 was chosen such that the human participants were able to observe, on average, one feature per target. Other experiments were similarly performed by considering a range of parameters that spanned task difficulty levels across participants and treasure hunt types.

### 4.3 Sensory deprivation

**FIGURE 4:**
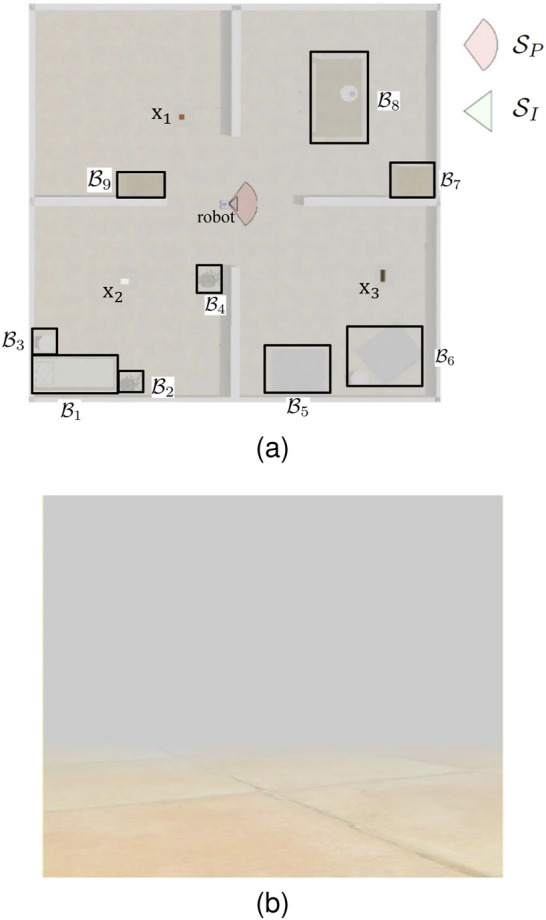
Top view visibility conditions of the unknown workspace **(A)** and first-person view of poor visibility condition **(B)** due to fog.

As explained in Section 2, information-gathering agents were not provided a map of the workspace W *a priori*, and, instead, were required to obtain information about target and obstacle positions and geometries by means of a passive on-board sensor (e.g., camera or LIDAR) with FOV $S_{P}$ as shown in Figure 4A. From the definition of set visibility region (Definition 2.4), for a subset S⊆{1,2,…,r} of target indices, the set visibility region $V_{S}\subseteq C_{\text{free}}$ contains all targets in S visible to passive sensor with FOV $S_{P}$ . A globally optimal solution to treasure hunt problem (Equations 1–7) with respect to a subset of targets S is feasible if and only if $V_{S}\neq \emptyset$ .

In parallel to the human studies in Section 3.2, robot sensory deprivation was introduced by simulating/producing fog in the workspace, thereby reducing the FOV radius to approximately 1 m, in a 20 m × 20 m robot workspace. A fog environment is simulated inside the Webots® environment as shown in Figure 4, thereby reducing the camera’s ability (Figure 4B) to view targets inside the sensor $S_{P}$ . As a result, $V_{S}=\emptyset$ even when there are |S|=2 targets, indicating that a globally optimal solution is infeasible. Consequently, optimal strategies typically fail under sensory deprivation due to lack of target information. Using the methods presented in the next section, human strategies for modulating between satisficing and optimizing strategies are first learned from data and, then, generalized to autonomous robots, as shown in Section 8. Satisficing strategies are aimed at overcoming this difficulty, and use local information to explore the environment and visit targets.

## 5 Mathematical modeling of human passive satisficing strategies

**FIGURE 5:**
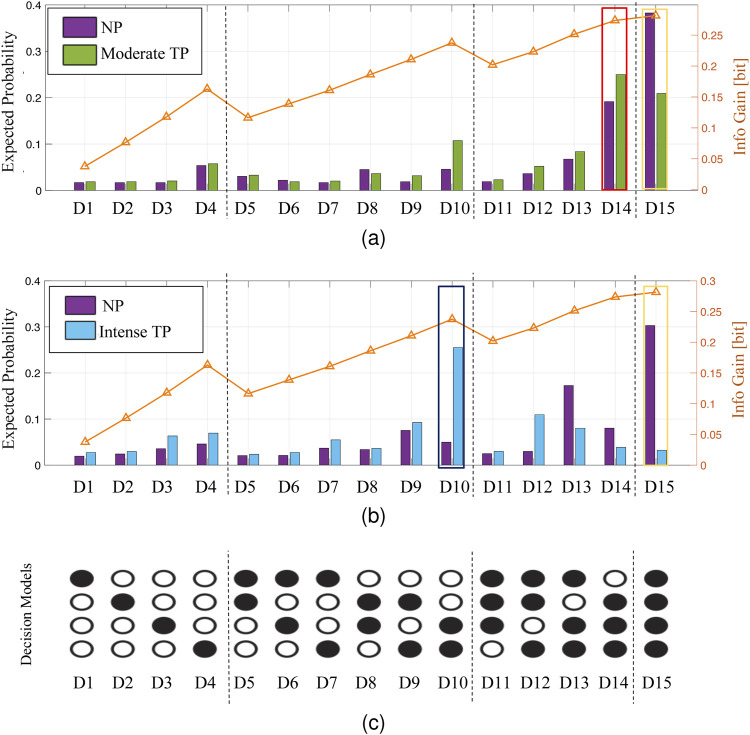
Human data analysis results for the moderate TP experiment **(A)** and the intense TP experiment **(B)** with the enumeration of decision models **(C)**.

Previous work by the authors showed that human participants drop less informative features to meet pressing time deadlines that do not allow them to complete the tasks optimally (Oh et al., 2016). The analysis of data obtained from the moderate TP experiment (Figure 5A) and intense TP experiment (Figure 5B) reveals similar interesting findings regarding human decision-making under different time pressure conditions. Under the no TP condition, the most probable decision model selected by human participants (indicated by the yellow contour for D15 in Figures 5A, B) utilizes all four features and aims at maximizing information value. However, under moderate TP, the most probable decision model selected by human participants (indicated by a red box in Figure 5A) uses only three features and has lower information value than the no TP condition. As time pressure becomes the most stringent in the intense TP, the most probable decision model selected by human participants (indicated by a dark blue box in Figure 5B) uses only two features and exhibits even lower information value than observed in the previous two time pressure conditions. Figure 5C shows all possible decision models (i.e. features combinations) that a participant can use to make an inferential decision. These results demonstrate the trade-offs made by human participants among time pressure, model complexity, and information value. As time pressure increases, individuals adaptively opt for simpler decision models with fewer features, and sacrificed information value to meet the decision deadline, thus reflecting the cognitive adaptation of human participants in response to time constraints.

### 5.1 Passive satisficing decision heuristic propositions

Inspired by human participants’ satisficing behavior indicated by the data analysis above, this paper develops three heuristic decision models, which accommodate varying levels of time pressure and adaptively select a subset of information-significant features to solve the inferential decision making problems. For simplicity and based on experimental evidence, it was assumed that observed features were error free.

#### 5.1.1 Discounted cumulative probability gain (ProbGain)

The heuristic is designed to incorporate two aspects of behaviors observed from human data. First, the heuristic encourages the use of features that provide high information value for decision-making. By summing up the information value of each feature, the heuristic prioritizes the features that contribute the most to evidence accumulation. Second, the heuristic also considers the cost of using multiple features in terms of processing time. By applying a higher discount to models with more features, the heuristic discourages excessive cost on time that might lead to violation of time constraints.

For an inferential decision-making problem with sorted p observed features ${\left\{x_{j}\right\}}_{j=1}^{p}$ according to the information value $v_{\text{ProbGain}}\left(x_{j}\right)$ in descending order, where $v_{\text{ProbGain}}\left(x_{j}\right)$ representing the increase in information value with respect to the maximum a-posterior rule

$$v_{\text{ProbGain}}\left(x_{j}\right)=\max_{y\in Y}p\left(Y=y\;|\;x_{j}\right)-\max_{y\in Y}p\left(Y=y\right)$$

Let $\left\{x_{1},x_{2},\ldots ,x_{i}\right\}$ represent a subset of observed features that contains the first (i) most informative features with respect to $v_{\text{ProbGain}}\left(x_{j}\right)$ , where $t_{T}$ is the allowable time to make a classification decision, and the discount factor γ∈(0,1) is defined to be a function of $t_{T}$ in order to represent the penalty induced by time pressure. Then, the heuristic strategy can be modeled as follows,

$$H_{\text{ProbGain}}\left(t_{T},{\left\{x_{j}\right\}}_{j=1}^{p}\right)={\mathrm{arg max}}_{i}\left\{\gamma {\left(t_{T}\right)}^{i}\overset{i}{\underset{j=1}{\sum }}v_{\text{ProbGain}}\left(x_{j}\right)\right\}$$

where,

$$\gamma \left(t_{T}\right)=\exp\left(-\frac{\lambda }{t_{T}}\right)$$

and, thus, λ may be used to represent the extent to which the discount γ is applied to the cue information value.

#### 5.1.2 Discounted log-odds ratio (LogOdds)

Log odds ratio plays a central role in classical algorithms like logistic regression (Bishop and Nasrabadi, 2006), and represents the “confidence” of making a inferential decision. The update of log odds ratio with respect to a “new feature” is through direct summation, thus taking advantage of the feature independence and arriving at fast evidence accumulation. Furthermore, the use of log odds ratio in the context of time pressure is slightly modified such that a discount is applied with inclusion of an additional feature to penalize the feature usage because of time pressure. By combining the benefits of direct summation for fast evidence accumulation and the discount for time pressure as inspired from human behavior, the heuristic based on log odds ratio can make efficient decisions by considering the most relevant features under time constraints.

For an inferential decision-making problem with sorted p observed features ${\left\{x_{j}\right\}}_{j=1}^{p}$ according to the information value $\;|\;v_{\mathrm{ProbGain}}\left(x_{j}\right)\;|\;$ in descending order, where $\;|\;v_{\mathrm{ProbGain}}\left(x_{j}\right)\;|\;$ represents the log odds ratio of observed features $x_{j}$ . Then, the heuristic strategy can be modeled as follows,

$$H_{\text{LogOdds}}\left(t_{T},{\left\{x_{j}\right\}}_{j=1}^{p}\right)={\mathrm{arg max}}_{i}\left\{\gamma {\left(t_{T}\right)}^{i}\;|\;v_{0}+\overset{i}{\underset{j=1}{\sum }}v_{\mathrm{ProbGain}}\left(x_{j}\right)\;|\;\right\}$$

where

$$v_{I}\left(x_{j}\right)=log\left(p\left(x_{j}\;|\;y_{1}\right)\right)-log\left(p\left(x_{j}\;|\;y_{2}\right)\right)$$

$$v_{0}=log\left(p\left(Y=y_{1}\right)\right)-log\left(p\left(Y=y_{2}\right)\right)$$

#### 5.1.3 Information free feature number discounting (InfoFree)

The previous two feature selection heuristics are both based on comparison: multiple candidate sets of features are evaluated and compared, and the heuristics select the one with the best trade-off between information value and processing time cost. A simpler heuristic is proposed to avoid comparisons and reduces the computation burden, while still showing the behavior that dropping less informative features due to time pressure observed from human participants.

Sort the p features according to the information value $v_{I}\left(x_{j}\right)$ in descending order as $x_{1},x_{2},\ldots ,x_{p}$ , and a subset of the first i most informative features refers to as $\left\{x_{1},x_{2},\ldots ,x_{i}\right\}$ . The heuristic strategy is as follows

$$H_{\text{InfoFree}}\left(t_{T}\right)=\left\lceil p\exp\left(-\frac{\lambda }{t_{T}}\right)\right\rceil$$

The outputs of the three heuristics are the numbers of features to be fed into the model $P\left(Y_{i},X_{i,1},\ldots ,X_{i,n}\right)$ to make an inference decision. Some mathematical properties (e.g., convergence and monotonicity) of the three proposed heuristic strategies are presented in Supplementary Appendix SA1.

### 5.2 Model fit test against human data

**FIGURE 6:**
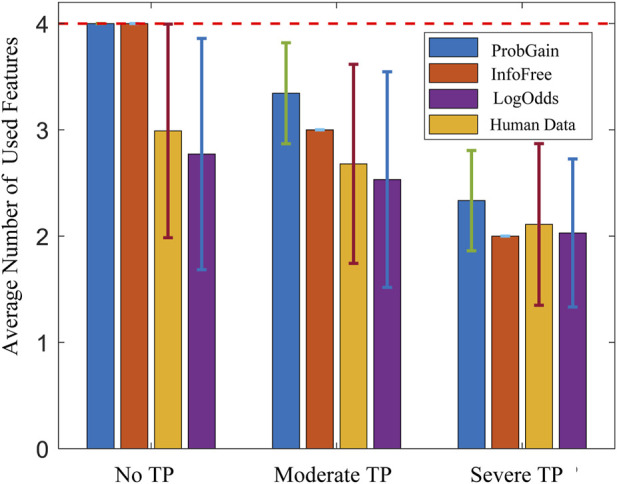
Mean and standard deviation of the number of used features of three heuristic strategies and the human strategy under three time pressure levels.

The model fit tests against human data of the three proposed time-adaptive heuristics are under three time pressure levels, with the time constraints scaled to ensure comparability between human experiments and heuristic tests. The results, as shown in Figure 6, indicate two major observations. First, as time pressure increases, all three strategies utilize fewer features, thus demonstrating their adaptability to time constraints and mirroring the behavior observed in human participants. Second, among the three strategies, $H_{\text{LogOdds}}$ exhibits the closest average number of features and standard deviation to the human data across all time pressure conditions. Consequently, $H_{\text{LogOdds}}$ is the heuristic strategy that best matches the human data among the three proposed strategies.

## 6 Autonomous robot applications of passive satisficing strategies

The effectiveness of the human passive satisficing strategies modeled in the previous section, namely, the three heuristics denoted by $H_{\text{ProbGain}}$ , $H_{\text{LogOdds}}$ , and $H_{\text{InfoFree}}$ , was tested on an autonomous robot making inferential decisions on the well-established database known as car evaluation dataset (Dua and Graff, 2017). This dataset, containing 1,728 samples, is chosen over other benchmark problems because its size is comparable to the database used for modeling human heuristics and is characterized by six possibly redundant features, which allows for the ability to adaptively select a subset of features to infer the target class. The performance of the three heuristics is compared against that of a naïve Bayes classifier, referred to as “Bayes optimal” herein, which utilizes all available features for decision-making.

The car evaluation dataset records the cars’ acceptability, on the basis of six features and originally four classes. The four classes are merged into two. A training set of 1,228 samples is used to learn the conditional probability tables (CPTs), ensuring equal priors for both classes. After learning the CPTs, 500 samples are used to test the classification performance of the heuristics and the naïve Bayes classifier. The tests are conducted under three conditions: no TP, moderate TP, and intense TP.

**FIGURE 7:**
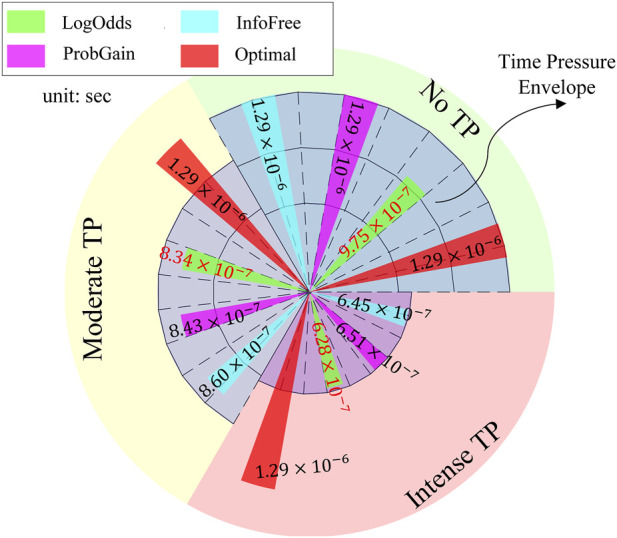
Processing time (unit: sec) of three time-adaptive heuristics and the “Bayes optimal” strategy.

The experiments are performed on a digital computer using MATLAB R2019b on an AMD Ryzen 9 3900X processor. The processing times of the strategies are depicted in Figure 7. If a heuristic’s processing time falls within the time pressure envelope (blue area), the time constraints are considered satisfied. The no TP condition provides sufficient time for all heuristics to utilize all features for decision-making. The moderate TP condition allows for 75% of the time available in the no TP condition, whereas the intense TP condition allows for 50% of the time available in the no TP condition. All three heuristics are observed to satisfy the time constraints across all time pressure conditions.

**FIGURE 8:**
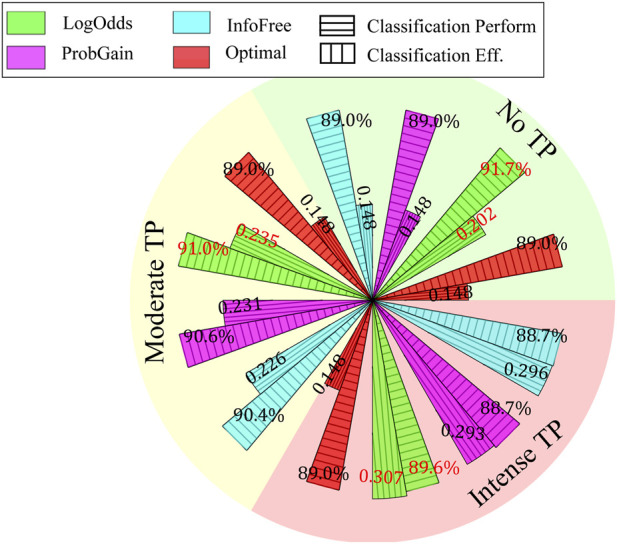
Classification performance and efficiency of three time-adaptive heuristics under three time pressure conditions.

The classification performance and efficiency of the three time-adaptive strategies is plotted in Figure 8. $H_{\text{LogOdds}}$ outperforms the other three strategies on this dataset, and its performance deteriorates as time pressure increases. Under moderate TP, the three time-adaptive strategies use fewer features but achieve better classification performance than Bayes optimal. This finding exemplifies the less-can-be-more effect (Gigerenzer and Gaissmaier, 2011). The classification efficiency measures the average contribution of each feature to the classification performance. Bayes optimal displays the lowest efficiency, because it utilizes all features for all time pressure conditions, whereas $H_{\text{LogOdds}}$ exhibits the highest efficiency among the three heuristics across all time pressure conditions.

## 7 Mathematical modeling of human active satisficing strategies

In the active satisficing experiments, human participants face pressures due to (unmodelled) information cost (money) and sensory deprivation (fog pressure). These pressures prevent the participants from performing the test and action decisions optimally. The data analysis results for the information cost pressure, as described in Section 7.1, reveal that the test decisions and action decisions are coupled. The pressure on test decisions affect the action decisions made by the participants. The data analysis of the sensory deprivation (fog pressure) does not incorporate existing decision-making models, such as Ziebart et al. (2008); Levine et al. (2011); Ghahramani (2006); Puterman (1990), because the human participants perceive very limited information, thus violating the assumptions underlying these models. Instead, a set of decision rules are extracted in the form of heuristics from the human participants data from inspection. These heuristics capture the decision-making strategies used by the participants under sensory deprivation (fog pressure).

### 7.1 Information cost (money) pressure

Previous studies showed that, when information cost was present, humans used a single good reason strategy (e.g., take-the-best) in larger proportion than compensatory strategies, which integrated all available features, to make decisions (Dieckmann and Rieskamp, 2007); and information cost induced humans to optimize decision criteria and shift strategies to save cost on inferior features (Bröder, 2003). This section analyzes the characteristics of human decision behavior under information cost pressure compared with no pressure condition.

Based on the classic “treasure hunt” problem formulation for active perception (Ferrari and Wettergren, 2021), the goals of action and test decisions are expressed through three objectives, namely, information value or benefit (B) , information cost (J) , and distance travelled (D) . Hence, optimal strategies are typically assumed to maximize a weighted sum of the three objectives, i.e.,

$$V=\overset{T}{\underset{k=0}{\sum }}\omega_{B}B\left(t_{k}\right)-\omega_{D}D\left(t_{k}\right)-\omega_{J}J\left(t_{k}\right)$$ (8)

where, the weights $\omega_{B}$ , $\omega_{D}$ , and $\omega_{J}$ represent the relative importance of the corresponding objectives.

Upon entering the study, human participants are instructed to solve the treasure hunt problem by maximizing the number of treasures found using minimum time (distance) and money. Therefore, it can be assumed that human participants also seek to maximize the objective function in (23), using their personal criteria for relative importance and decision strategy. Since the mathematical form of the chosen objectives is unknown, upon trial completion the averaged weights utilized by human participants are estimated using the Maximum Entropy Inverse Reinforcement Learning algorithm, adopted from Ziebart et al. (2008). The learned weights can then be used to understand the effects of money pressure on human decision behaviors, as follows. The two indices, $I_{IG}=\omega_{B}/\omega_{D}$ and $I_{IC}=\omega_{B}/\omega_{J}$ , are obtained from the ratios of the three averaged weights and, thus, reflect the priorities underlying human decisions and behaviors. The first index, $I_{IG}$ , referred to as information-value attempt index, measures the willingness of human participants to trade travel distance in favor of increased information value. The second index, $I_{IC}$ , referred to as information-cost parsimony index, measures the willingness of human participants to spend “money” in favor of increased information value.

**FIGURE 9:**
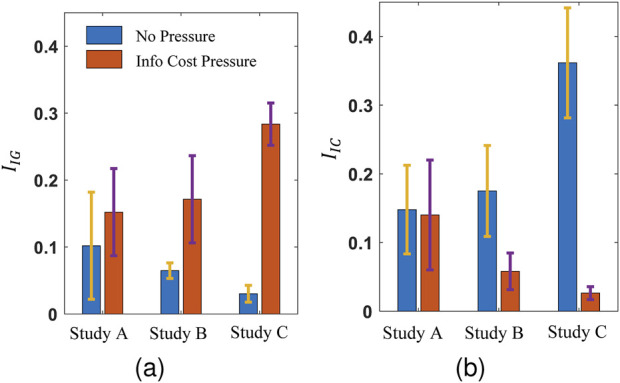
**(A)** The information value attempt index, $I_{IG}$ , and **(B)** information-cost parsimony index $I_{IC}$ are shown for high-performance human participants under two pressure conditions.

**FIGURE 10:**
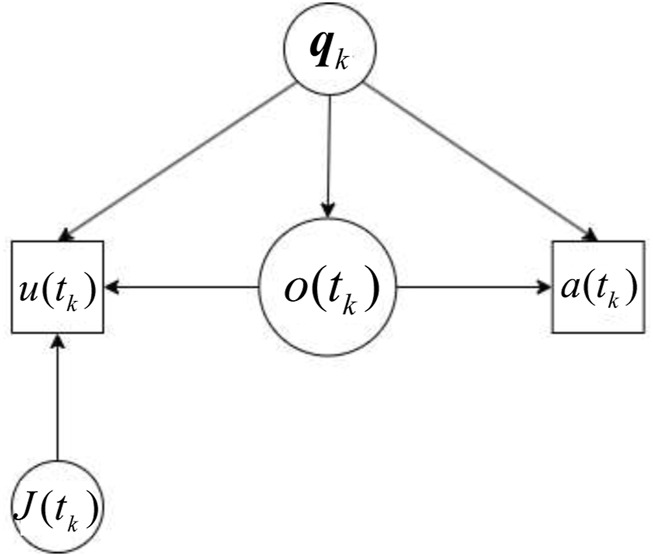
The intra-slice DBN that models human decision behavior.

The analysis of human experiment data, shown in Figure 9, indicates that, under information cost (money) pressure, human participants are willing to travel longer distances to acquire information of high value $\left(↑I_{IG}\right)$ . However, they are less willing to incur costs $\left(↓I_{IC}\right)$ for information value, thus suggesting a tendency to be more conservative in spending resources for information acquisition. Furthermore, assuming no other utility (goal) is associated with human states or actions, the causal relationships underlying human decisions may be modeled using dynamic Bayesian networks (DBNs) learned from the human trials. The DBN intra-slice structure, shown in Figure 10, uses nodes to represent the human participants’ states $q_{k}$ , action decision $a\left(t_{k}\right)$ , test decision $u\left(t_{k}\right)$ , the set of visible targets $o\left(t_{k}\right)$ at time $t_{k}$ , and the “money”(information cost) already spent $J\left(t_{k}\right)$ . The intra-slice variables capture the relevant information for decision-making at a specific time slice, learning both arcs and parameters from human data.

Once the DBN description of human decisions is obtained, the inter-slice structure may be used to understand how observations influence subsequent action and test decisions. The key question is: in how many time slices does an observation $o\left(t_{k}\right)$ influence decision-making? To determine the appropriate inter-slice structure, this paper conducts a series of hypothesis tests to assess the conformity of various models against the human decision data. Supplementary Figure S3 presents the results of these hypothesis tests. Each data point represents a p -value that evaluates the null hypothesis: “model i+1 does not fit the human data significantly better than model i ”. The models are defined according to the number of time slices in which an observation influences decisions. If the p -value is smaller than the significance level α , the null hypothesis is rejected, thus indicating that the subsequent model fits the data better than the previous one.

According to the results plotted in Supplementary Figure S3, under the no pressure condition, an observation $o\left(t_{k}\right)$ influences one subsequent decision. However, under the information cost(money) pressure, an observation $o\left(t_{k}\right)$ influences nine subsequent decisions. This finding suggests that the influence of observations extends over a longer time horizon under information cost(money) pressure than in the no pressure condition.

### 7.2 Sensory deprivation (fog pressure)

The introduction of sensory deprivation (fog pressure) in the environment poses two main difficulties for human participants during navigation. First, fog limits the visibility range, thus hindering human participants’ capability of locating targets and being aware of obstacles. Second, fog impairs spatial awareness, thus hindering human participants’ ability to accurately perceive their own position within the workspace.

**FIGURE 11:**
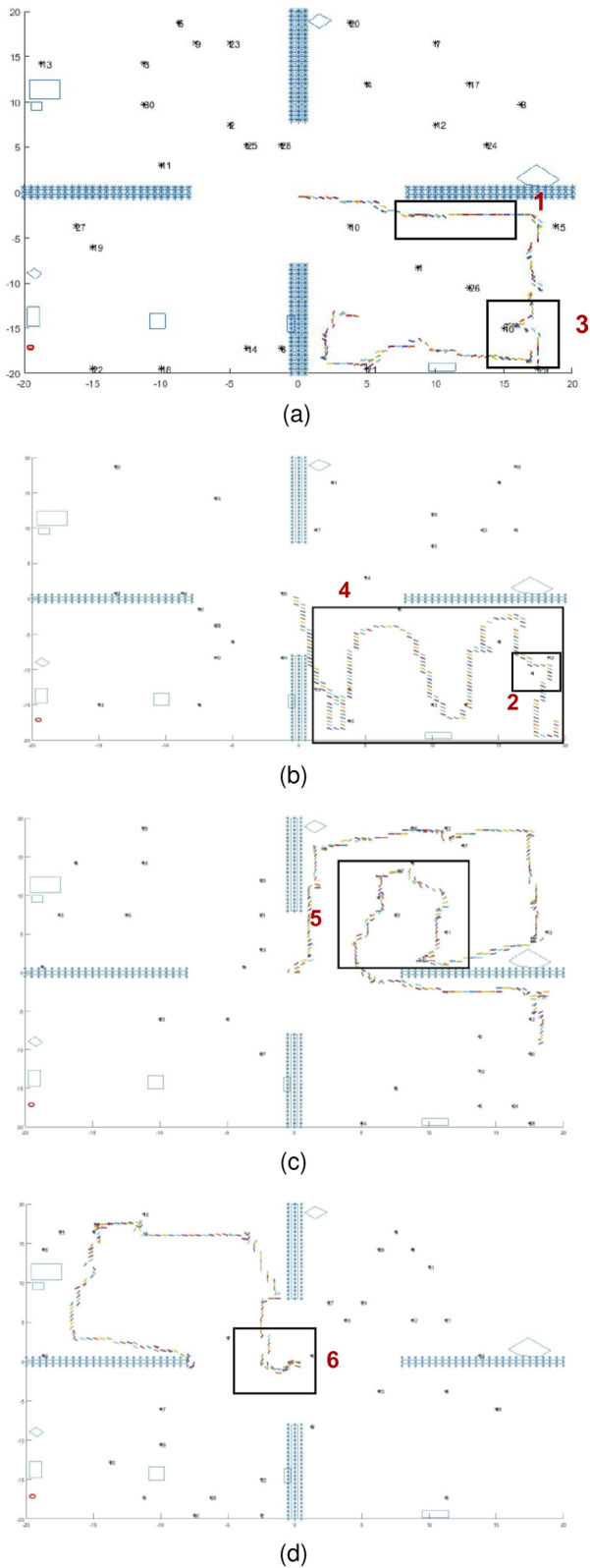
The human behavior patterns in a fog environment, which demonstrate wall following **(A)**, area coverage **(B)**, strategy switching **(C)**, and random walk **(D)** behaviors.

In situations in which target and obstacle information is scarcely accessible and uncertainties are difficult to model, human participants were found to use local information to navigate the workspace, observe features, and classify all targets in their FOVs (Gigerenzer and Gaissmaier, 2011; Dieckmann and Rieskamp, 2007). By analyzing the human decision data collected through the active satisficing experiments described in Section 3, significant behavioral patterns shared by the top human performers can be summarized by the following six behavioral patterns exemplified by the sample studies plotted in Figure 11:

1. When participants enter an area and no targets are immediately visible, they follow the walls or obstacles detected in the workspace (Figure 11A).
2. When participants detect multiple targets, they pursue targets one by one, prioritizing them by proximity (Figure 11B).
3. While following a wall or obstacle, if participants detect a target, they will deviate from their original path and pursue the target, and may then return to their previous “wall/obstacle follow” path after performing classification (Figure 11A).
4. Upon entering an enclosed area (e.g., room), participants may engage in a strategy of covering the entire room (Figure 11B).
5. After walking along a wall or obstacle for some time without encountering any targets, participants are likely to switch to a different exploratory strategy (Figure 11C).
6. In the absence of any visible targets, participants may exhibit random walking behavior (Figure 11D).

Detailed analysis of the above behavioral patterns (omitted for brevity) showed that the following three underlying incentives drive human participants in the presence of fog pressure:

• Frugal: Human participants exhibit tendencies to avoid repeated visitations. Navigating along walls or obstacles helps participants localize themselves by using walls or obstacles as reference points.

• Greedy: Human participants demonstrate a strong motivation to find targets and engage with them. After a target is detected, participants pursue it and interact with it immediately.

• Adaptive: Human participants display adaptability by using multiple strategies for exploring the workspace. These strategies include “wall/obstacle following,” “area coverage,” and “random walk.” Participants can switch among these strategies according to the effectiveness of their current approach in finding targets.

Based on these findings, a new algorithm referred to as AdaptiveSwitch (Algorithm 1) was developed to emulate humans’ ability to transition between the three heuristics when sensory deprivation prevents the implementation of optimizing strategies. The three exploratory heuristics consist of wall/obstacle following $\left(\pi_{1}\right)$ , area coverage $\left(\pi_{2}\right)$ , and random walk $\left(\pi_{3}\right)$ . The probability of executing each heuristic is referred to as $\Pi ={\left[b_{1},b_{2},b_{3}\right]}^{T}$ , where $b_{i}$ represents the probability of executing $\pi_{i}$ . The index g indicates the exploratory policy being executed, and k represents the number of steps taken while executing a policy. The maximum number of steps before updating the distribution Π is K . The policy for interacting with targets is $\pi_{I}\left(u\left(t_{k}\right)\;|\;q_{k},o\left(t_{k}\right)\right)$ , and the policy for pursuing a target is $\pi_{P}\left(a\left(t_{k}\right)\;|\;q_{k},o\left(t_{k}\right)\right)$ .

Algorithm 1 AdaptiveSwitch.

1: $\Pi ={\left[b_{1},b_{2},b_{3}\right]}^{T}$

2: k=0,g=0

3: **while** ( $t_{k}\leq t_{T}\;\vee$ not all targets are classified) **do**
4:  **if** $\exists x_{j}\in S_{I}\left(q_{k}\right)$ **then**
5: $\pi_{I}\left(u\left(t_{k}\right)\;|\;q_{k},o\left(t_{k}\right)\right)$

6:  **else**
7:   **if** $o\left(t_{k}\right)\neq \emptyset$ **then**
8: $\pi_{P}\left(a\left(t_{k}\right)\;|\;q_{k},o\left(t_{k}\right)\right)$

9: k=0,g=0

10:   **else**
11:    **if** g>0 ∧ k≤K **then**
12: $\pi_{g}\left(a\left(t_{k}\right)\;|\;q_{k},o\left(t_{k}\right)\right)$

13: k=k+1

14:    **else**
15:     **if** k≥K **then**
16: Π[g]=γ*Π[g]

17:     **else**
18:      **if** not closed to wall **then**
19: Π[1]=0

20:      **else**
21: $\Pi \left[1\right]=\beta \left(b_{1}+b_{2}+b_{3}\right)$

22:      **end if**
23:     **end if**
24: Π= normalize (Π)

25: g∼Π

26:    **end if**
27:   **end if**
28:  **end if**
29: **end while**

As shown in Algorithm 1, the greediness of the heuristic strategy (lines 4–9) captures the behaviors in which participants interact with targets if possible (line 4) and pursue a target if it is visible (line 7). If no targets are visible and the maximum exploratory step K is not exceeded, the current exploratory heuristic continues to be executed (lines 11–13). The adaptiveness of the three exploratory heuristics is shown in lines 15–22. If the current exploratory heuristic is executed for more than K steps, its probability of execution is discounted (line 16). The probability of executing the “wall/obstacle following” heuristic increases β > 1.0 if the participant is close to a wall/obstacle; otherwise this heuristic is disabled (lines 19–21).

**FIGURE 12:**
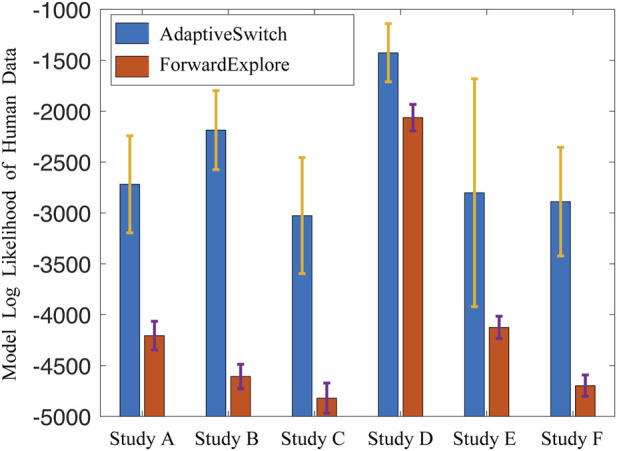
Averaged model log likelihood of AdaptiveSwitch and ForwardExplore in six human studies.

After learning the parameters from the human data, the AdaptiveSwitch algorithm was compared to another hypothesized switching logic referred to as ForwardExplore in which participants predominantly move forward with a high probability and turn with a small probability or when encountering an obstacle. In order to determine which switching logic best captured human behaviors, the log likelihood of AdaptiveSwitch and ForwardExplore was computed using the human data from the active satisficing experiment involving six participants. The results plotted in Figure 12 show that the log likelihood of AdaptiveSwitch is greater than that of ForwardExplore across all human experiment trials. This finding suggests that AdaptiveSwitch aligns more closely with the observed human strategies than ForwardExplore and, therefore, was implemented in the robot studies described in the next section.

## 8 Autonomous robot applications of active satisficing strategies

Two key contributions of this paper are the applications of the modeled human strategies on a robot, and the comparison of optimal strategies and the modeled human strategies in pressure conditions, under which optimization is infeasible. For simplicity, the preferred sensing directions of $S_{P}$ and $S_{I}$ are assumed to be fixed with respect to the robot platform. Therefore, the state vector for a robot reduces to $q={\left[x\;y\;\theta \right]}^{T}$ , where the orientation of the robot platform θ also represents the preferred sensing directions. Both sensor FOVs are modeled by sectors with angle-of-view $\zeta_{1},\zeta_{2}\in \left[0,2\pi \right)$ and radii $r_{1},r_{2}>0$ . The two FOVs share the same apex and their bisectors coincide with each other.

### 8.1 Information cost (money) pressure

The introduction of information cost increases the complexity of planning test decisions. In the absence of information cost, a greedy policy that observes all available features for any target is considered “optimal”, because it collects all information value without any cost. However, when information cost is taken into account, a longer planning horizon for test decisions becomes crucial to effectively allocate the budget for observing features of all targets. This paper implements two existing robot planners, PRM and cell decomposition, to solve the treasure hunt problem in an identical workspace, initial conditions, and target layouts faced by human participants in the active satisficing treasure hunt experiment. The objective function Equation 8 is maximized by using these methods. Unlike existing approaches (Ferrari and Cai, 2009; Cai and Ferrari, 2009; Zhang et al., 2009) that solve the original version of the treasure hunt problem as described in (Ferrari and Wettergren, 2021), the developed planners handle the problem without pre-specification of the final robot configuration. Consequently, the search space increases exponentially, thus rendering label-correcting algorithms (Bertsekas, 2012) no longer applicable. Additionally, unlike previous methods that solely optimize the objective with respect to the path, the developed planners consider the constraint on the number of observed features due to information cost pressure. The number of observed features thus becomes a decision variable with a long planning horizon. To solve the problem, the developed planners use PRM and cell decomposition techniques to generate graphs representing the workspace (Ferrari and Wettergren, 2021). The Dijkstra algorithm is used to compute the shortest path between targets. Furthermore, an MINLP algorithm is used to determine the optimal number of observed features and the visitation sequence of the targets.

#### 8.1.1 Performance comparison with human strategies

The performance of the optimal strategies known as PRM and cell decomposition is compared to that of human strategies in Supplementary Figure S4. It can be seen that, under information cost (money) pressure, the path and number of observed features per target are optimized using a linear combination of three objectives. Letting τ denote the planned path (as defined in (LaValle, 2006)), four performance metrics are used for evaluation and comparison, i.e.,: path efficiency $\eta_{P}=1/D\left(\tau \right)$ $\left[m^{-1}\right]$ ; information gathering efficiency $\eta_{B}=B\left(\tau \right)/D\left(\tau \right)$ [bit/m] ; measurement productivity $\eta_{J}=B\left(\tau \right)/J\left(\tau \right)$ [bit]; and classification performance N=N(τ) (with higher values indicating higher performance). Six case studies are examined. One case study comprises of three different experiment layouts. The optimal strategies and the human participants have no prior knowledge of the target positions and initial features, and all environmental information is obtained from FOV $S_{P}$ . The results, shown in Supplementary Figure S4, indicate that the two optimal strategies consistently outperform the human strategy across all four performance metrics. The performance envelopes of the optimal strategies are outside of the performance of the human strategy, thus indicating their superiority.

The finding that the optimal strategies outperform human strategies is unsurprising, because information cost (money) pressure imposes a constraint on only the expenditure of measurement resources, which can be effectively modeled mathematically. The finding suggests that under information cost (money) pressure, near-optimal strategies can make better decisions than human strategies.

### 8.2 Sensory deprivation (fog pressure)

An extensive series of tests are conducted to evaluate the effectiveness of AdaptiveSwitch (Section 7.) under sensory deprivation(fog) conditions and compare it with other strategies. These tests comprise of 118 simulations and physical experiments, encompassing various levels of uncertainty. The challenges posed by fog in robot planning are twofold. First, fog obstructs the robot’s ability to detect targets and obstacles by using onboard sensors such as cameras, thus making long-horizon optimization-based planning nearly impossible. Second, fog complicates the task of self-localization for the robot with respect to the entire map, although short-term localization can rely on inertial measurement units. Three test groups are described as follows:

#### 8.2.1 Performance comparison tests inside human experiment workspace

AdaptiveSwitch is applied to robots operating in the same workspace and target layouts used in the active satisficing human experiments (Section 3.), described in Figures 3, 4. Using these eighteen environments, the performance of hypothesized human strategies, AdaptiveSwitch and ForwardExplore, was compared to that of existing robot strategies (cell decomposition and PRM). One important metric used to evaluate a strategy’s capability to search for targets in fog conditions is the number of classified targets: $N_{v}$ . As shown by the quantitative comparison in Supplementary Figure S5A and Supplementary Figure S5B, under sensory deprivation (fog) the optimal strategies face difficulties in moving and classifying targets because of the lack of information on target and obstacle layout. Here, the distance travelled and classification performance are plotted by averaging the results of extensive simulations, along with the standard deviation (bars in Supplementary Figures S5A, S5B). In contrast, both the human strategies and AdaptiveSwitch are able to explore the unknown environment, even if at times they do not capture target information through $S_{P}$ . In particular, AdaptiveSwitch achieves slightly higher target classification rates and shorter travel distances than the observed human strategies.

#### 8.2.2 Generalized performance comparison

**FIGURE 13:**
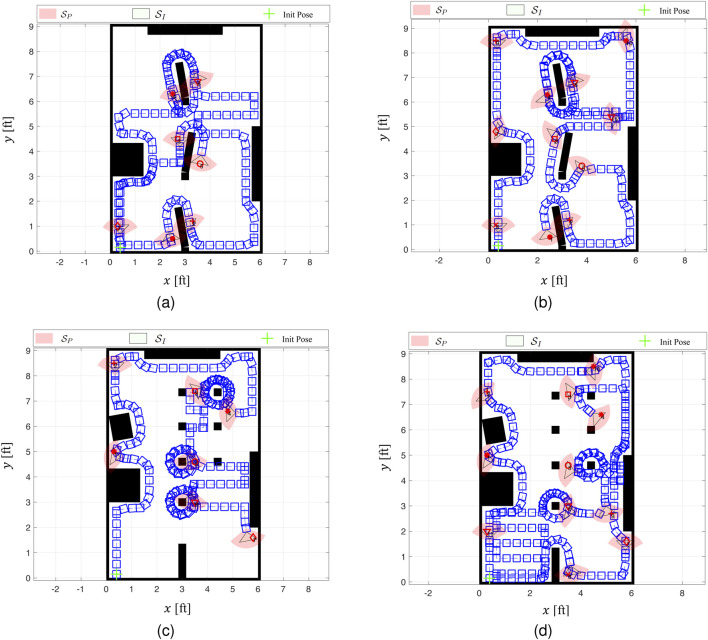
Four workspace in MATLAB® simulations and AdaptiveSwitch trajectories for case studies $\left(a\right)-\left(d\right)$ : with 7 targets plus 9 obstacles **(A)**, 11 targets plus 9 obstacles **(B)**, 7 targets plus 12 obstacles **(C)**, and 11 targets plus 12 obstacles **(D)**.

In order to demonstrate the generalizability of the human-inspired strategy AdaptiveSwitch to robot applications, extensive comparative studies were performed using new workspaces and target layouts, different from those used in human experiments. In order to fully assess the performance and generalizability of AdaptiveSwitch, the sensor range was also varied to investigate the influence of sensor modalities and characteristics. Extensive simulations were conducted in ${MATLAB}^{Ⓡ}$ using four newly designed workspaces and corresponding target layouts (Figure 13). For evaluation purposes, these additional simulations considered fixed FOV geometries and assumed no missed detections or false alarms, as well as perfect target feature recognition.

**FIGURE 14:**
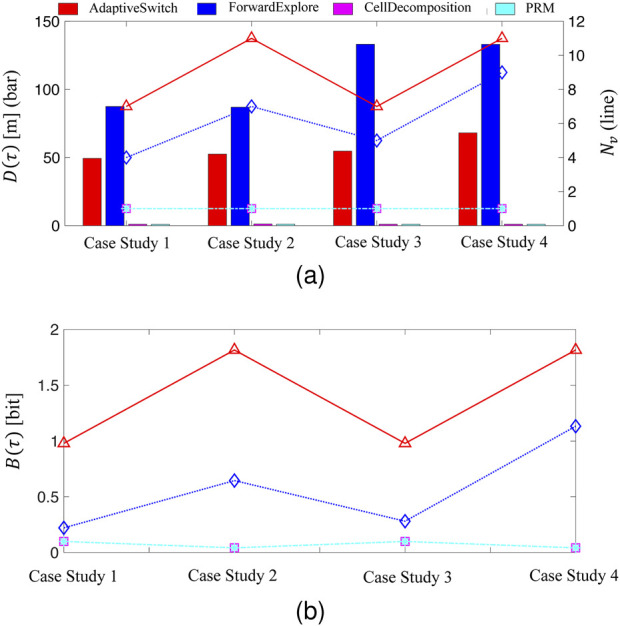
**(A)**Number classified targets and travel distance **(B)**information gain for two heuristic strategies and two existing robot strategies in four case studies.

As part of this comparison, ForwardExplore and the two existing robot strategies, cell decomposition and PRM, are also implemented for comparison. Due to the limitations posed by fog and limited sensing capabilities, the performance in terms of travel distance, D(τ) , and classification, $\left(N_{v}\right)$ , is significantly hindered, as shown by the averaged values plotted in Figure 14A on the left (histogram bar) and right (line) vertical axis, respectively. Robots implementing AdaptiveSwitch outperform those implementing other strategies in terms of the number of correctly classified targets, because they are able to explore the workspace even when no targets were visible.

Additionally, AdaptiveSwitch is more efficient than ForwardExplore in terms of travel distance. By adapting its exploration strategy and leveraging the combination of three simple heuristics, AdaptiveSwitch is able to classify more targets while traveling shorter distances. Consequently, higher information value B(τ) than that with both ForwardExplore and existing robot strategies is observed across all four case studies (Figure 14B). These findings highlight the effectiveness of the AdaptiveSwitch in navigating foggy environments and its superiority to existing robot strategies and the ForwardExplore in terms of information gathering and travel efficiency.

##### 8.2.2.1 Simulations with artificial fog

Two new workspaces are designed in Webots® as shown in Supplementary Figure S6. The performance of AdaptiveSwitch and its standalone heuristics for the two workspaces is shown in Tables 2, 3. The comparison reveals the substantial advantage of AdaptiveSwitch. In both workspace scenarios, as shown in Tables 2, 3, AdaptiveSwitch outperforms its standalone heuristics by successfully finding and classifying all targets within the given simulation time upper bound. In contrast, the standalone heuristics are unable to achieve this level of performance. AdaptiveSwitch not only visits and classifies all targets, but also accomplishes the tasks within shorter travel distances than the standalone heuristics. Therefore, AdaptiveSwitch exhibits higher target visitation efficiency $\left(\eta_{v}\right)$ which is calculated as the ratio of the number of classified targets to the travel distance $\left(N_{v}/D\left(\tau \right)\right)$ . The target visitation efficiency of AdaptiveSwitch is at least twice higher than that of the standalone heuristics thanks to the combination of multiple, simple heuristics. In contrast, when used in a stand-alone fashion, the same heuristics may become trapped in ineffective “moving patterns”, struggling to perform in certain areas of the workspace.

**TABLE 2:** Performance comparison of AdaptiveSwitch and Standalone heuristics in Webots®: Workspace A.

Performance metrics	Heuristic strategies		
	AdaptiveSwitch	RandomWalk	AreaCoverage
Travel distance, D(τ) [m]	86.19	164.87	224.18
Number of classified targets, $N_{v}$	7/7	7/7	3/7
Target visitation efficiency, $\eta_{v}\;\left[{\text{m}}^{-1}\right]$	0.0812	0.0425	0.0134
Travel distance, D(τ) [m]	148.98	291.69	246.38
Number of classified targets, $N_{v}$	13/13	11/13	6/13
Target visitation efficiency, $\eta_{v}\;\left[{\text{m}}^{-1}\right]$	0.0873	0.0377	0.0244
Travel distance, D(τ) [m]	159.97	236.86	205.78
Number of classified targets, $N_{v}$	15/15	11/15	8/15
Target visitation efficiency, $\eta_{v}\;\left[{\text{m}}^{-1}\right]$	0.0938	0.0464	0.0389

**TABLE 3:** Performance comparison of AdaptiveSwitch and Standalone heuristics in Webots®: Workspace B.

Performance metrics	Heuristic strategies		
	AdaptiveSwitch	RandomWalk	AreaCoverage
Travel distance, D(τ) [m]	122.86	218.72	265.49
Number of classified targets, $N_{v}$	7/7	5/7	5/7
Target visitation efficiency, $\eta_{v}\;\left[{\text{m}}^{-1}\right]$	0.0570	0.0229	0.0188
Travel distance, D(τ) [m]	122.57	219.49	234.70
Number of classified targets, $N_{v}$	13/13	10/13	7/13
Target visitation efficiency, $\eta_{v}\;\left[{\text{m}}^{-1}\right]$	0.0873	0.0456	0.0298
Travel distance: D(τ) [m]	129.19	226.57	216.25
Number of classified targets, $N_{v}$	15/15	12/15	8/15
Target visitation efficiency, $\eta_{v}\;\left[{\text{m}}^{-1}\right]$	0.1161	0.0530	0.0370

#### 8.2.3 Physical experiment tests in real fog environment

To handle real-world uncertainties that are not adequately modeled in simulations, this paper conducts physical experiments to test the AdaptiveSwitch. These uncertainties include factors such as the robot’s initial position and orientation, target miss detection and false alarms, depth measurement errors, and control disturbances. In addition, the fog models available in Webots®, are relatively simple and do not provide a wide range of possibilities for simulating the degrading effects of fog on target detection and classification performance. Consequently, this paper performs physical experiments to better capture the complexities and uncertainties associated with real-world conditions.

The physical experiments use the ROSbot2.0 robot equipped with an RGB-D camera as the primary sensor. The YOLOv3 object detection algorithm, the best applicable at the time of these studies, was implemented to detect targets of interest (e.g., an apple, watermelon, orange, basketball, computer, book, cardboard box, and wooden box) identical to those in human experiments. Training images for the YOLOv3 were obtained in fog-free environments, in order to later test the robot’s ability to cope with unseen conditions (fog pressure) in real time.

**FIGURE 15:**
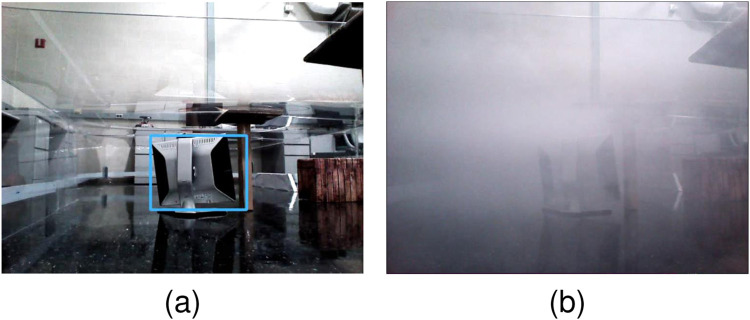
Object detection results **(A)** in clear and **(B)** fog conditions.

As shown in Figure 15, the YOLOv3 algorithm successfully detects the existence of the target “computer” when the environment is clear, as shown in Figure 15A. However, when fog is present, as illustrated in Figure 15B, the algorithm fails to detect the target. This result demonstrates the degrading effect on the performance of target detection algorithms.

In the physical experiments conducted with ROSbot2.0 (Husarion, 2018), AdaptiveSwitch and ForwardExplore are implemented to test their performance in an environment with fog. A plastic box is constructed with dimensions 10′0″ x 6′0″ x 1′8″ in order to create the foggy environment. The box is designed to contain different layouts of obstacles and targets, capturing various aspects of a “treasure hunt” scenario, such as target density and target view angles. Each heuristic strategy is tested five times in each layout, considering all the uncertainties described earlier. The travel distances in the physical experiments are measured in inertial measurement unit.

**FIGURE 16:**
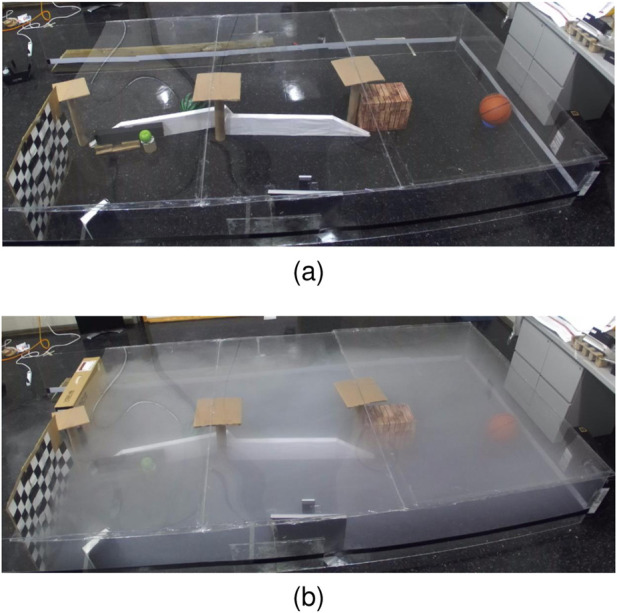
The first workspace and target layout for the physical experiment under **(A)** clear and **(B)** fog condition.

**FIGURE 17:**
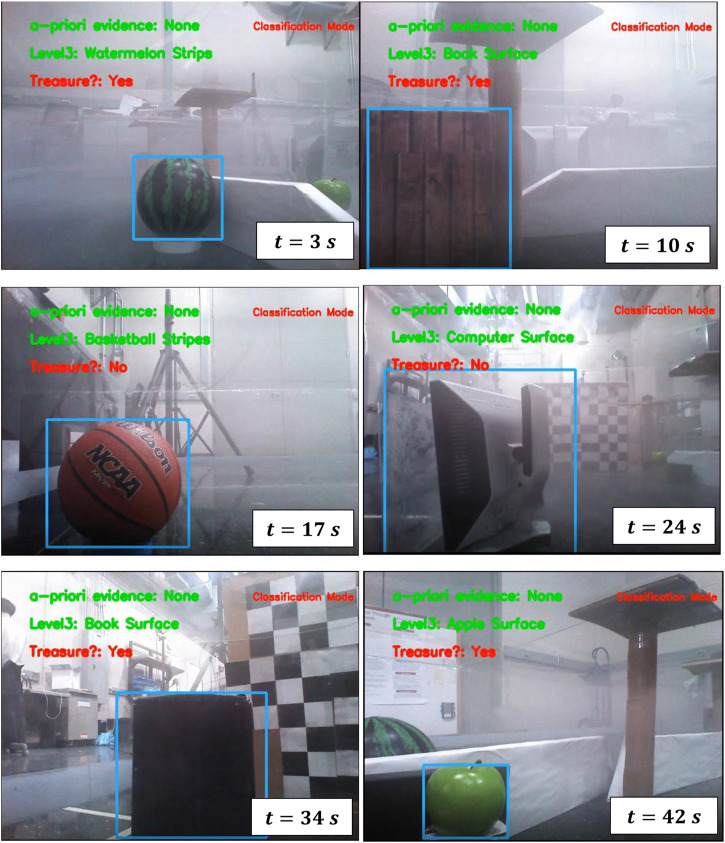
Target visitation sequence of AdaptiveSwitch in the first workspace.

The first layout (Figure 16) is comprised of six targets, i.e.,: a watermelon, wooden box, basketball, book, apple, and computer. The target visitation sequences of AdaptiveSwitch along the path are depicted in Figure 17, showing the robot’s trajectory and the order in which the targets are visited. The performance of the two strategies is summarized in Table 4, as evaluated according to three aspects: travel distance D(τ) , correct target feature classifications, and information gathering efficiency $\eta_{B}$ . These metrics assess the quality of the strategies’ action and test decisions.

**TABLE 4:** Performance Comparison of Heuristic Strategies in target layout 1.

Performance metrics	Heuristic strategies	
	AdaptiveSwitch	ForwardExplore
Number of classified targets, $N_{v}$	6/6	6/6
Travel distance, D(τ) [m]	6.43 ± 0.90	8.38 ± 2.07
Correct target feature classifications	13.40 ± 1.82	12.40 ± 1.95
Info gathering efficiency, $\eta_{B}\;\left[\text{bit/m}\right]$	0.155 ± 0.023	0.090 ± 0.018

**FIGURE 18:**
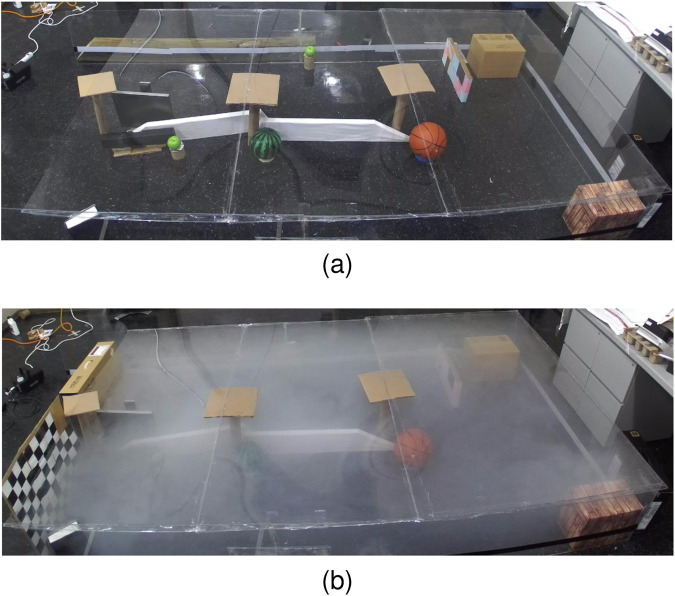
The second workspace and target layout for the physical experiment under **(A)** clear and **(B)** fog condition.

**FIGURE 19:**
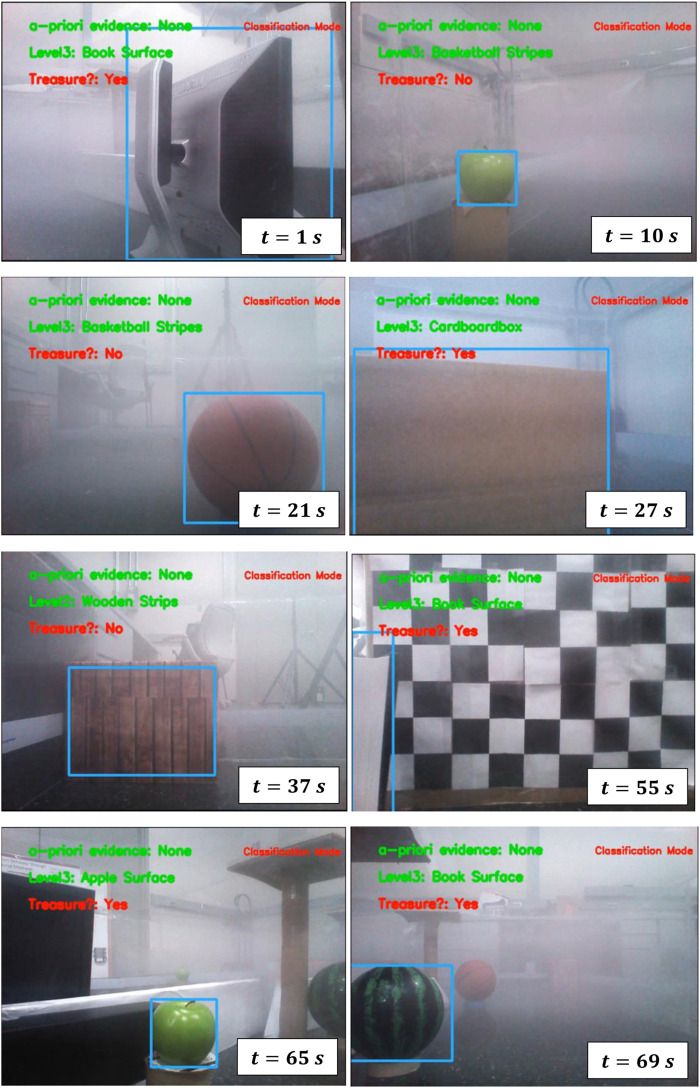
Target visitation sequence of AdaptiveSwitch in the second workspace.

The second layout (Figure 18) contains eight targets: a watermelon, wooden box, basketball, book, computer, cardboard box, and two apples. The obstacles layout is also changed with respect to the first layout: the cardboard box is placed in a “corner” and is visible from only one direction, thus increasing the difficulty of detecting this target. This layout enables a case study in which the targets are more crowded than in the first layout. The mobile robot first-person-views of AdaptiveSwitch along the path are demonstrated in Figure 19, and the performance is shown in Supplementary Table S1.

**FIGURE 20:**
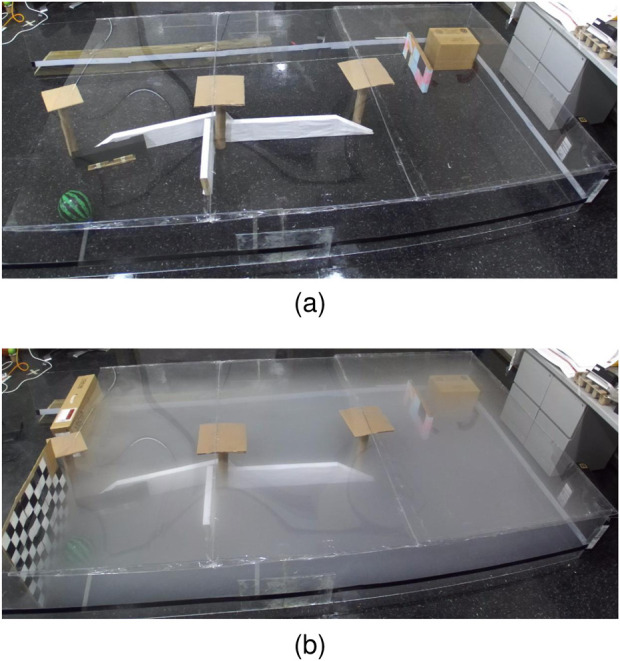
The third workspace and target layout for the physical experiment under **(A)** clear and **(B)** fog condition.

**FIGURE 21:**
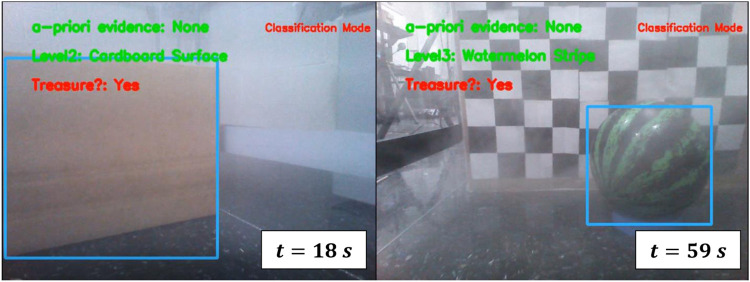
Target visitation sequence of AdaptiveSwitch in the third workspace.

The third layout (Figure 20) contains two targets: a cardboard box, and a watermelon. Note that having fewer targets does not necessarily make the problem easier, because the difficulty in target search in fog comes from how to navigate when no target is in the FOV. This layout intentionally makes the problem “difficult”, because it “hides” two targets behind the walls. The mobile robot first-person-views of AdaptiveSwitch along the path are demonstrated in Figure 21, and the performance is shown in Supplementary Table S2. The videos for all physical experiments (AdaptiveSwitch and ForwardExplore in three layouts) are accessible through the link in (Chen, 2021).

According to the performance summaries in Table 4, Supplementary Tables S1, S2, both AdaptiveSwitch and ForwardExplore are capable of visiting and classifying all targets in the three layouts under real-world uncertainties. However, AdaptiveSwitch demonstrates several advantages over ForwardExplore:

1. The average travel distance of AdaptiveSwitch is 30.33%, 59.93%, and 56.02% more efficient than ForwardExplore in the three workspaces, respectively. This finding indicates that AdaptiveSwitch is able to search target with a shorter travel distance than ForwardExplore.
2. The target feature classification performance of AdaptiveSwitch is slightly better than that of ForwardExplore, with improvements of 8.06%, 17.11%, and 4.16% in the three workspace, respectively. One possible explanation for these results is that the “obstacle follow” and “area coverage” heuristics in AdaptiveSwitch cause the robot’s body to be parallel to obstacles during classification of target features, thus ensuring that the targets are the major part of the robot’s first-person view and make them relatively easier to classify. In contrast, ForwardExplore does not always lead the robot body to be parallel to obstacles during classification, thereby sometimes allowing obstacles to dominate the robot’s first-person view and decreasing the target classification performance.

## 9 Summary and conclusion

This paper presents novel satisficing solutions that modulate between near-optimal and heuristics to solve satisficing treasure hunt problem under environment pressures. These proposed solutions are derived from human decision data collected through both passive and active satisficing experiments. The ultimate goal is to apply these satisficing solutions to autonomous robots. The modeled passive satisficing strategies adaptively select target features to be entered in measurement model based on a given time pressure. The idea behind this approach is the human participants behavior that dropping less informative features for inference in order to meet the decision deadline. The results show that the modeled passive satisficing strategies outperform the “optimal” strategy that always use all available features for inference in terms of classification performance and significantly reduce the complexity of target feature search compared with exhaustive search.

Regarding the active satisficing strategies, the strategy that deals with information cost formulates an optimization problem with the hard constraint imposed by information cost. This approach is taken because the information cost constraint doesn’t fundamentally undermine the accuracy of the model of the world and the agent, and optimization still yield high-quality decisions. The results show that the strategy outperforms human participants across several key metrics (e.g., travel distance and measurement productivity, etc.). However, under sensory deprivation, the knowledge of the world is severely compromised, and thus decisions produced by optimization is risky or even no longer feasible, which is also demonstrated through experiments in this paper. The modeled human strategies named AdaptiveSwitch shows the ability to use local information and navigate in foggy environment by using heuristics derived from humans. The results also show that the AdaptiveSwitch can adapt to varying workspaces with different obstacle layouts, target density, etc., beyond the workspace used in the active satisficing experiments. Finally, AdaptiveSwitch is implemented on a physical robot and conducts satisificing treasure hunt with actual fog, which demonstrates the ability to deal with real-life uncertainties in both perception and action.

Overall, the proposed satisficing strategies comprise of a toolbox, which can be readily deployed on a robot in order to address different real-life environment pressures encountered during the mission. These strategies provide solutions to scenarios characterized by time limitations, constraints on available resources (e.g., fuel or energy), and adverse weathers such as fog or heavy rain.

## Data Availability

The raw data supporting the conclusions of this article will be made available by the authors, without undue reservation.
