# Supplementary material for: Heuristic satisficing inferential decision making in human and robot active perception
Source: Front Robot AI. 2024 Nov 12;11:1384609. doi: 10.3389/frobt.2024.1384609 (PMC11589672; doi:10.3389/frobt.2024.1384609)
Supplement: Supplementary file 1 [file Supplementaryfile1.pdf]

Appendix A
Mathematical Properties of Heuristics Under Time Pressure

A. Discounted Cumulative Probability Gain (ProbGain)

Proposition 1. A sufficient condition for *ProbGain* to use all p features is that the allowable time t_T to make a decision satisfies:

$$t_T \geq \frac{\lambda p}{\ln(1+\frac{\alpha}{p})} \tag{1}$$

where $\alpha = v_I(x_p)/v_I(x_1)$ is the ratio of information values between the least informative feature and the most informative feature.

Proposition 2. A sufficient condition for *ProbGain* to use 1 (the least possible number of features to use) feature is that the allowable time to make a decision t_T satisfies

$$t_T \leq \frac{\lambda}{\ln(p)} \tag{2}$$

Proposition 3.

Monotonicity with respect to allowable time t_T for a classification task with features $\{x_i\}_{i=1}^p$, $H_{\text{ProbGain}}(t_T, \{x_i\}_{i=1}^p)$ satisfies:

$$H_{\text{ProbGain}}(t_{T,2}, \{x_i\}_{i=1}^p) \geq H_{\text{ProbGain}}(t_{T,1}, \{x_i\}_{i=1}^p) \tag{3}$$

for $\forall t_{T,1}, t_{T,2},\ t_{T,2} > t_{T,1}$

Propositions 1 and 2 indicate the behavior of ProbGain under "extreme" conditions. Notably, proposition 1 shows that as the allowable time $t_T \geq \frac{\lambda p}{\ln(1+\frac{\alpha}{p})}$, the heuristic uses all features to make the classification decision (i.e., converges to the "optimal strategy," which uses all features to make a decision). In addition, according to Proposition 2, when the allowable time is too short ($t_T \leq \frac{\lambda}{\ln(p)}$), the heuristic only uses one feature (the least possible number of features to use) to make the decision. Proposition 3 shows the monotonicity of the heuristic with respect to allowable time t_T; as the allowable time increases, the heuristic uses monotonically more features to make a classification decision.

B. Discounted Log-odds Ratio (LogOdds)

This heuristic regards the log-odds ratio,

$$c_i = log\frac{p(Y = y_1 \mid x_1, ..., x_i)}{p(Y = y_2 \mid x_1, ..., x_i)}$$

on the basis of features in set $x_1, x_2, , ..., x_i$ represents the "confidence" of making the classification task. The greater is the value of $|c_i|$, the more confident is the classification decision. While one feature comes into consideration, an additional time-pressure dependent discount factor is imposed on the absolute value the log-odds ratio c_i of the features in set $\{x_1, x_2, , ..., x_i\}$. The heuristic selects the features under pressure according to the maximization of the product of the discount factors and the log-odds ratio. In this way, less informative features are dropped because of the discount factor. As the time pressure increases, the heuristic has a greater tendency to drop the features.

Proposition 4. A sufficient condition for *LogOdds* to use one feature is if the allowable time t_T to make a decision satisfies

$$t_T \leq \frac{\lambda}{\ln(1+\frac{p-1}{\mid 1+\beta \mid})} \tag{4}$$

where $\beta = v_0/v_I(x_1)$.

Proposition 5.

Monotonicity with respect to allowable time t_T: for an object with features $\{x_i\}_{i=1}^p$, $H_{\text{LogOdds}}(t_T, \{x_i\}_{i=1}^p)$ satisfies:

$$H_{\text{LogOdds}}(t_{T,2}, \{x_i\}_{i=1}^p) \geq H_{\text{LogOdds}}(t_{T,1}, \{x_i\}_{i=1}^p) \tag{5}$$

for $\forall t_{T,1}, t_{T,2},\ t_{T,2} > t_{T,1}$.

Note that unlike H_{ProbGain}, although H_{LogOdds} tends to use more features as time pressure is released, H_{LogOdds} does not necessarily use all p features when the time available t_T is greater than a certain threshold, because the value metric used in H_{LogOdds}: $|c_i| = |v_0 + \sum_{j=1}^{i} v_I(x_j)|$ is not monotonically increasing as the number of features to use i increases.

C. Information Free Feature Number Discounting (InfoFree)

After sorting the features in terms of the information value, the cut-off criterion of this heuristic is no longer dependent on the information value. Thus the allowable decision time t_T is the only argument for the heuristic. As $\exp(-\frac{\lambda}{t_T}) < 1, t_T > 0$, the number of features to use is always less than or equal to M and decreases exponentially when time pressure increases, and the parameter $\lambda > 0$ controls how much a time pressure is discounted. Given the monotonicity of the exponential function, H_{InfoFree} uses more features as time pressure is released and it uses all p features if the time available t_T is sufficiently large, and uses one feature if the time available t_T is sufficiently small.

TABLE I
Performance Comparison of Heuristic Strategies in target layout 2

Performance Metrics	Heuristic Strategies	
	AdaptiveSwitch	ForwardExplore
Number of classified targets, N_v	8/8	8/8
Travel distance, $D(\tau)$ [m]	**8.41 ± 0.46**	13.45 ± 2.10
Correct target feature classifications	**17.80 ± 1.10**	15.20 ± 1.64
Info gathering efficiency, η_B [bit/m]	**0.151 ± 0.008**	0.091 ± 0.016

TABLE II
Performance Comparison of Heuristic Strategies in target layout 3

Performance Metrics	Heuristic Strategies	
	AdaptiveSwitch	ForwardExplore
Number of classified targets, N_v	2/2	2/2
Travel distance, $D(\tau)$ [m]	**7.48 ± 0.465**	11.67 ± 1.37
Correct target feature classifications	**5.00 ± 1.00**	4.80 ± 1.64
Info gathering efficiency, η_B [bit/m]	**0.033 ± 0.003**	0.021 ± 0.002

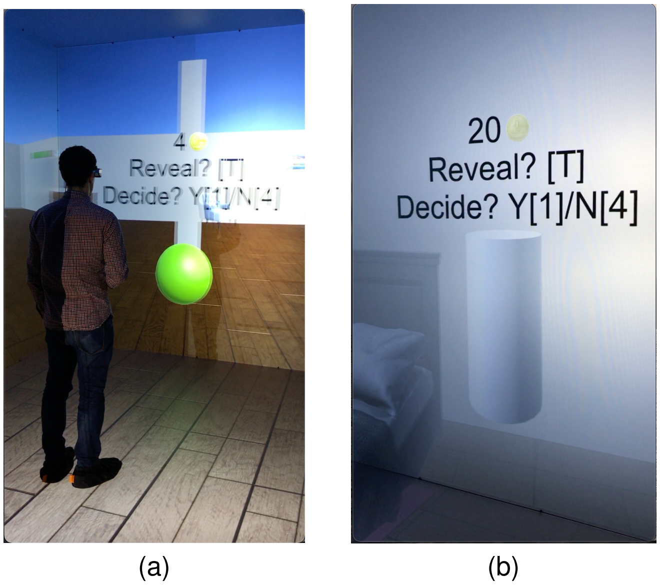

Fig. S1. Human participant solving treasure hunt problem under no pressures (a), and under sensory deprivation (fog) (b) in the Duke immersive Virtual Environment [1].

Feature dimension	Stimulus	Feature state 1	Feature state 2
Color		Blue	Red
Shape		Circle	Square
Contour		White	Black
Line Orientation		Horizontal	Vertical

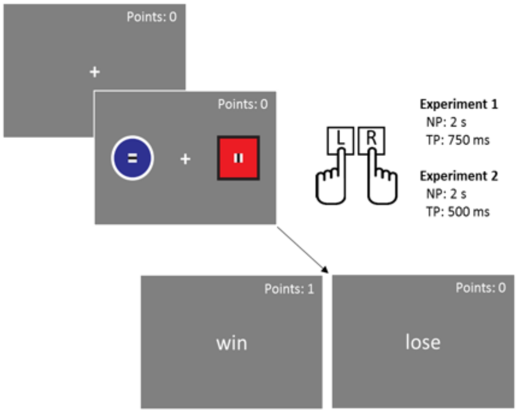

Fig. S2. Features and human display used for the passive satisficing experiment, where the result of "win" or "lose" was displayed only during the training phase.

TABLE S1
PERFORMANCE COMPARISON OF HEURISTIC STRATEGIES IN TARGET LAYOUT 2

Performance Metrics	Heuristic Strategies	
	AdaptiveSwitch	ForwardExplore
Number of classified targets, N_v	8/8	8/8
Travel distance, $D(\tau)$ [m]	**8.41 $\pm$ 0.46**	13.45 $\pm$ 2.10
Correct target feature classifications	**17.80 $\pm$ 1.10**	15.20 $\pm$ 1.64
Info gathering efficiency, η_B [bit/m]	**0.151 $\pm$ 0.008**	0.091 $\pm$ 0.016

TABLE S2
PERFORMANCE COMPARISON OF HEURISTIC STRATEGIES IN TARGET LAYOUT 3

Performance Metrics	Heuristic Strategies	
	AdaptiveSwitch	ForwardExplore
Number of classified targets, N_v	2/2	2/2
Travel distance, $D(\tau)$ [m]	**7.48 $\pm$ 0.465**	11.67 $\pm$ 1.37
Correct target feature classifications	**5.00 $\pm$ 1.00**	4.80 $\pm$ 1.64
Info gathering efficiency, η_B [bit/m]	**0.033 $\pm$ 0.003**	0.021 $\pm$ 0.002

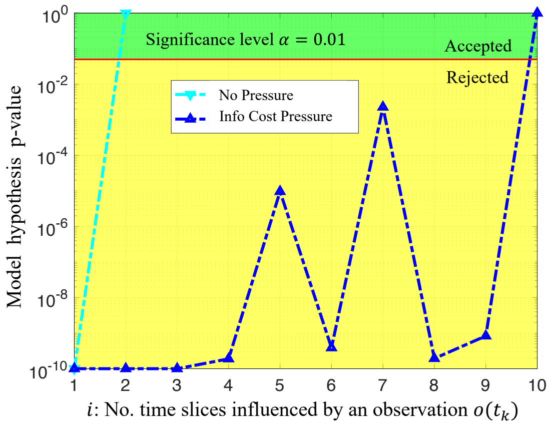

Fig. S3. DBN inter-slice structure hypothesis testing results

REFERENCES

[1] D. J. Zielinski, R. P. McMahan, W. Lu, and S. Ferrari, "Ml2vr: providing matlab users an easy transition to virtual reality and immersive interactivity," in *2013 IEEE Virtual Reality (VR)*. IEEE, 2013, pp. 83–84.

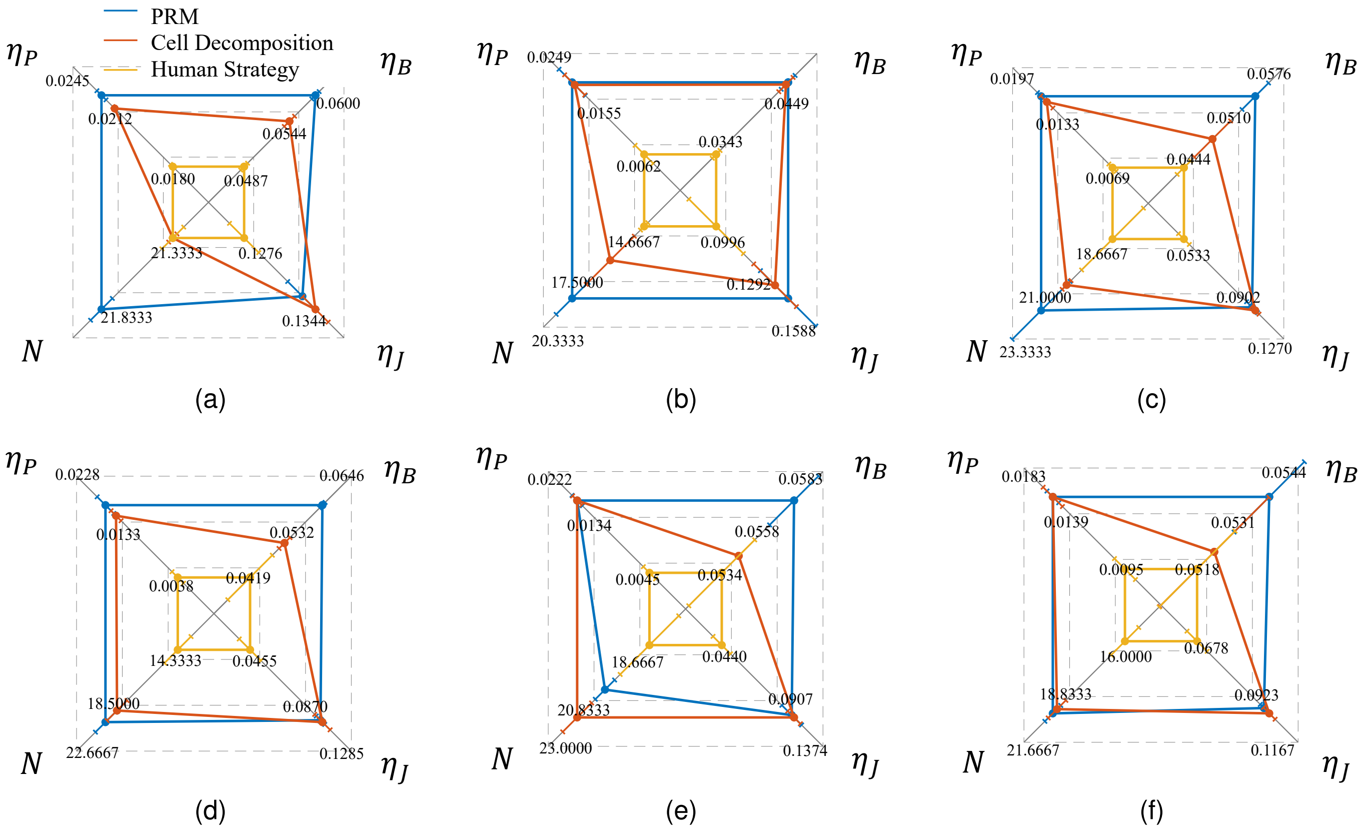

Fig. S4. Performance comparison of two optimal strategies and human strategy over six case studies (a)-(f).

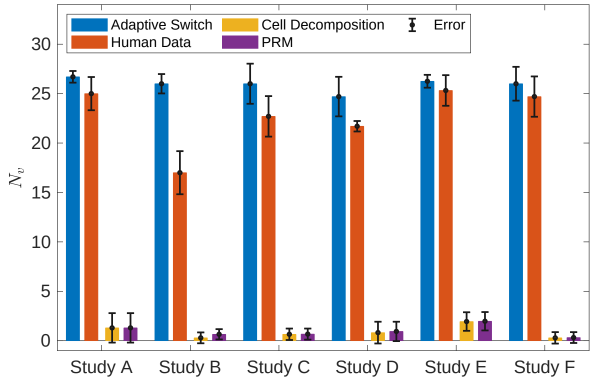

(a)

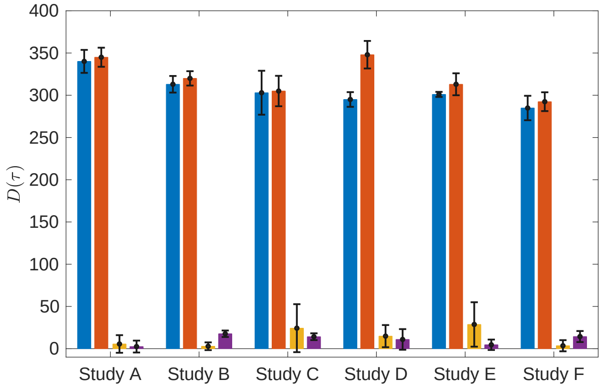

(b)

Fig. S5. (a) Number of classified targets and (b) travel distance of Adap-tiveSwitch optimal strategies and the human strategy, with average errors and standard deviations shown by superimposed vertical bars.

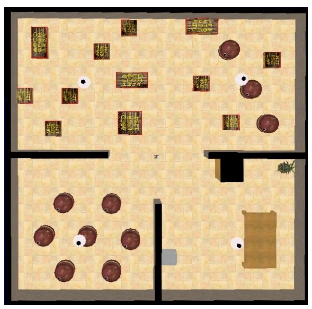

(a)

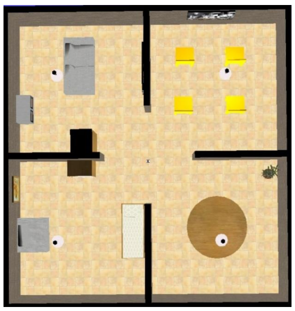

(b)

Fig. S6. New designs of workspace for heuristic strategy tests.
